# Genetic Differentiation, Isolation-by-Distance, and Metapopulation Dynamics of the Arizona Treefrog (*Hyla wrightorum*) in an Isolated Portion of Its Range

**DOI:** 10.1371/journal.pone.0160655

**Published:** 2016-08-09

**Authors:** Meryl C. Mims, Lorenz Hauser, Caren S. Goldberg, Julian D. Olden

**Affiliations:** 1 School of Aquatic and Fishery Sciences, University of Washington, Seattle, Washington, United States of America; 2 School of the Environment, Washington State University, Pullman, Washington, United States of America; National Cheng Kung University, TAIWAN

## Abstract

Population attributes such as diversity, connectivity, and structure are important components of understanding species persistence and vulnerability to extinction. *Hyla wrightorum*, the Arizona treefrog, is native to the southwestern United States and Mexico, and an isolated group of populations exists in the Huachuca Mountains and Canelo Hills (HMCH) of southeastern Arizona, USA. Due to concerns about declining observations of the species within the isolated HMCH portion of its range, the HMCH group is currently a candidate for federal protection under the U.S. Endangered Species Act. We present results of a genetic study examining population diversity, structure, and connectivity within the HMCH region. We sampled DNA from *H*. *wrightorum* larvae and adults from ten distinct locations, 8 of which were breeding sites and 4 of which were previously undescribed localities for the species. We developed and genotyped 17 polymorphic microsatellite loci and quantified genetic diversity, population differentiation, and landscape influences on population genetic structure. We found evidence of larger than expected effective population sizes, significant genetic differentiation between populations, and evidence of distance being the primary driver of genetic structure of populations with some influence of slope and canopy cover. We found little evidence of recent genetic bottlenecks, and individual-based analyses indicate admixture between populations despite significant genetic differentiation. These patterns may indicate that the breeding sites within the Huachuca Mountains constitute a metapopulation. We suggest that the HMCH region may contain larger and more connected breeding populations than previously understood, but the dynamics of this system and the limited geographic extent of the HMCH group justify current concern for the persistence of the species in this region. Efforts to ensure availability of high-quality breeding habitats and control for local threats such as effects of invasive predators may be critical to the persistence of these unique populations of *H*. *wrightorum*.

## Introduction

Effective conservation of species vulnerable to extinction throughout some or all of their range requires knowledge of population attributes such as diversity, connectivity, and structure [1]. For decades, population genetic approaches have helped successfully identify scenarios that may compromise the health, resilience, or persistence of a species and its populations [2]. Population genetic approaches have proven particularly useful in evaluating the status of amphibian species [3], many of which are declining globally due to threats including habitat loss, invasive species, disease, and climate change [4–5].

**Fig 1:**
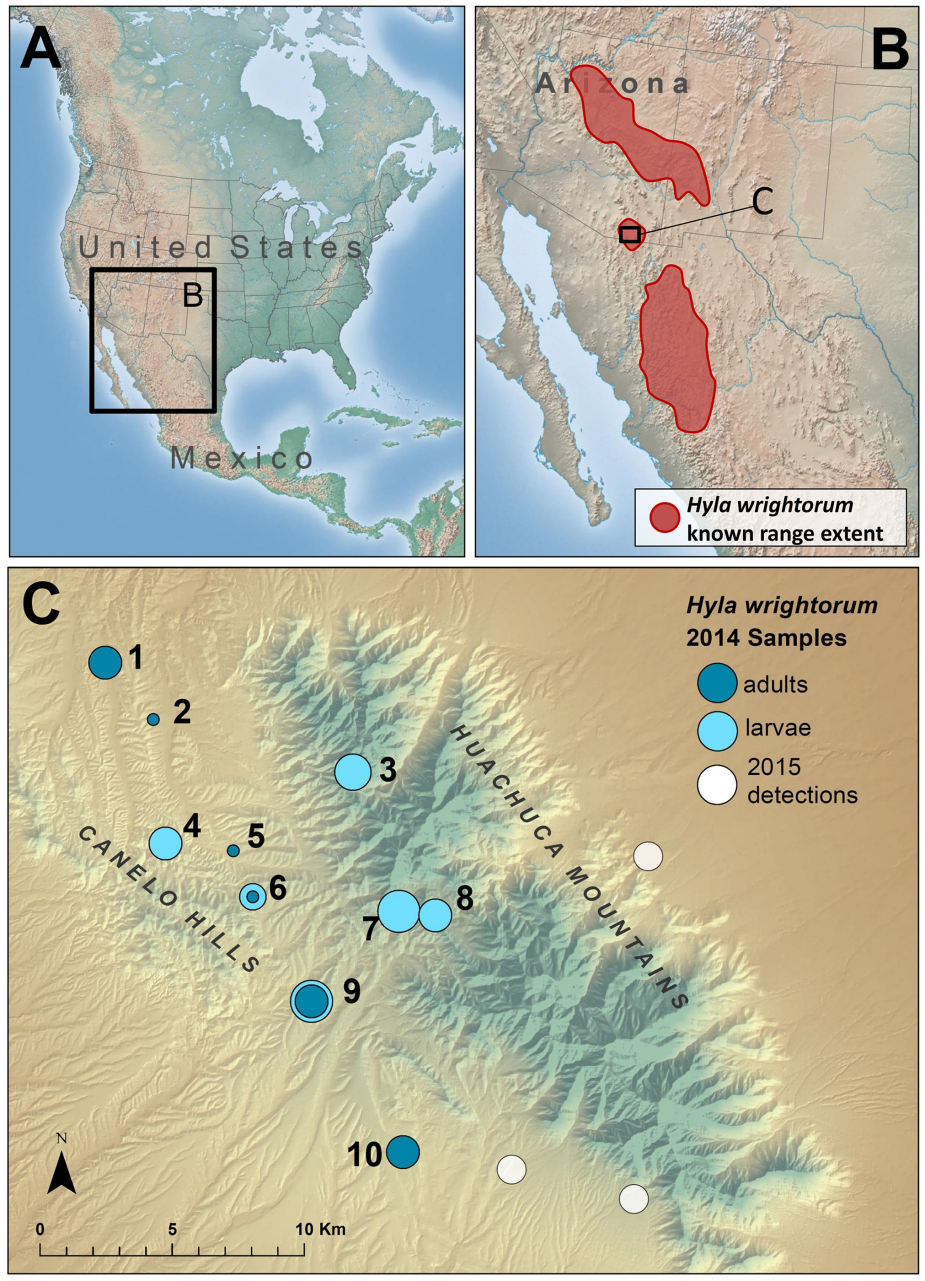
*Hyla wrightorum* range map and sampling locations. The known range extent of *H*. *wrightorum* [6, 9] (A, B), and *H*. *wrightorum* sampling locations in the Huachuca Mountains and Canelo Hills region (C). Symbols indicate 2014 sampled locations (blue) or unsampled locations at which *H*. *wrightorum* were observed in 2015 (white). Symbols for sampled locations are proportional to sample size, and color indicates life stage of sampled individuals (dark blue = adults; light blue = larvae). Population numbers are shown in black font, and information for each sampling location included in Table 1.

*Hyla wrightorum* (Taylor, 1938), the Arizona treefrog, is an anuran native to the southwestern United States and Mexico and is currently a candidate species for federal protection by the U.S. Fish and Wildlife Service (USFWS) within a subset of its range [6]. *Hyla wrightorum* is most commonly associated with streams, cienegas (wetlands), and manmade ponds, particularly during the summer monsoon season when intermittent pools are used for breeding [7–8]. The species’ range includes three disjunct regions, with the majority of the range occurring in two disparate regions in the United States and in Mexico. Spatially intermediate to these two regions is an isolated portion of the species’ range in the Huachuca Mountains and Canelo Hills (HMCH) region of southeastern Arizona, USA (Fig 1) [9–10]. This distinct portion of the species’ range is notable for its geographic isolation, and genetic evidence suggests that the HMCH region has been isolated from the other portions of the species’ range since the late Pleistocene [11].

*Hyla wrightorum* populations within the HMCH region are of conservation concern. In the past two decades, confirmed observations have been made at only half (8 of 16) of previously reported locations [6, 8]. The geographic range occupied by *H*. *wrightorum* in the HMCH is small compared to the larger two portions of its distribution (Fig 1), with known breeding sites occurring within an area no larger than 85 km^2^ [6]. Based on previous sampling efforts, HMCH population sizes were presumed to be small, with breeding choruses typically including 30 or fewer adults [6, 7]. Potential threats to local persistence of this species include disease [12] and predation by invasive American bullfrogs (*Lithobates catesbeianus* [13]). In addition, available breeding habitat is generally scarce in the region due to topographic constraints limiting suitable shallow breeding habitat [7]. Contemporary *H*. *wrightorum* breeding habitat in the HMCH region largely consists of manmade ponds originally built to provide water for livestock. Much of the region’s wetland habitat has been dewatered, and these “stock ponds” now serve as surrogate aquatic habitat for many native aquatic species. However, stock ponds also host non-native species, such as bullfrogs, which compete with and prey upon a host of native aquatic and terrestrial species [14]. Taken together, these factors contribute to the concern regarding the vulnerability of *H*. *wrightorum* populations in the HMCM region to regional processes such as dewatering, catastrophic fires, and other manmade disturbances likely to increase in frequency and intensity with climate change and increasing human development [15–17].

These concerns, coupled with the phenotypic, genetic, and geographic uniqueness of this group, have led to candidacy for federal protection of *H*. *wrightorum* in the HMCH region [6]. However, the status of populations in this isolated range is generally unknown. It is difficult to determine whether apparent absences over the last two decades at up to half of historically reported sites are due to true declines, natural population fluctuations, or failure to detect the species. Because *H*. *wrightorum* may form breeding choruses only one or a few nights in a given year, traditional census and survey efforts may fail to provide reliable information, particularly if populations are in fact in decline [18]. Genetic approaches provide promising alternatives to traditional census and survey efforts for evaluating population structure, size, and connectivity for many amphibians [3] and may offer valuable insight into the status of *H*. *wrightorum* in the Huachuca Mountains region.

We present results of a population genetic study for *H*. *wrightorum* in the HMCH region of southeastern Arizona. The objectives of our study were to address current knowledge gaps regarding population genetic diversity, population structure, and effective population sizes (*N*_*e*_) of this species in the region. To achieve this, we used a combination of population-level and individual-based approaches. Based on current knowledge of the species, we hypothesized small (< 30) effective population sizes for *H*. *wrightorum* in the HMCH region. This was based on both small observed breeding choruses for this species [6, 11] as well as recent reported estimates of *N*_*e*_ for a sympatric congener (*H*. *arenicolor*, average *N*_*e*_ = 30.7 [19]). We also searched for evidence of any recent genetic bottlenecks. Finally, we tested five hypotheses regarding the effects of landscape attributes on gene flow. First, if populations are sufficiently small, genetic drift may result in complete decoupling of genetic structure and spatial and landscape factors, producing isolated populations. This has been observed for the pond breeding salamander *Ambystoma mavortium stebbinsi*, sympatric to *H*. *wrightorum* in the Huachuca Mountains region [20]. Alternatively, an isolation-by-distance pattern may emerge in which populations in closer geographic proximity are genetically more similar than those farther apart [21]. We also tested three additional hypotheses of landscape effects: isolation-by-slope, in which steep topography inhibits gene flow; connectivity-by-canopy, in which gene flow is higher along more shaded areas that may provide more cover from predation or desiccation; and connectivity-by-stream in which gene flow is higher along riparian corridors. We conclude by discussing how the findings of this research can help inform management and conservation efforts for this species.

## Methods

### Sampling

Adult and larval *H*. *wrightorum* individuals were sampled during the summer monsoon season of 2014. We selected sampling locations using a combination of historical records of *H*. *wrightorum* occurrences as well as opportunistic visits to potential habitat (ponds and wetlands) in the known range of the species (Fig 1, Table 1). Sites were visited in the evenings, typically following thunderstorms, to maximize the chance of hearing breeding choruses. Sites were also surveyed during daylight hours using a combination of visual search and dip-netting. DNA was collected from each sampled individual via tail clip (larvae) or buccal swab (adult, following Goldberg et al. [22]). Tail clips were immediately stored with a desiccant (Drierite), and buccal swabs were immediately placed in a vial with buffer ATL from a DNeasy Blood & Tissue DNA extraction kit (QIAGEN). Tail clips were then kept at room temperature and buccal swabs at -10°C until DNA was extracted. Sampling protocol was approved by the University of Washington’s Institutional Animal Care and Use Committee (Protocol # 4172–03). All efforts were made to minimize animal handling time and suffering. All sampling was conducted on public lands, and sampling permits were obtained from the U.S. Forest Service (Special Use Permit SIE0150), Arizona Game and Fish Department (SP685479), and from the U.S. Army Garrison Fort Huachuca Environmental and Natural Resources Division (IMHU-PWB 200–1).

**Table 1:** *Hyla wrightorum* population attributes.

Pop	Sample size			Sample size, siblings removed		Genetic diversity					
	N_total_	N_adults_	N_larvae_	N_larvae_	N_total_	H_o_	H_e_	AR	N_e_	N_e, low_	N_e, high_
1	19	19	0	0	19	0.69	0.73	6.1	140.5	54.6	*Inf*.
3	43	0	43	30	30	0.67	0.69	5.8	277.9	88.0	*Inf*.
4	48	0	48	23	23	0.65	0.67	5.5	79.0	36.5	7999.7
6	34	6	28	14	20	0.69	0.73	6.0	51.7	31.4	120.0
7	50	0	50	37	37	0.70	0.74	5.7	109.1	56.8	526.0
8	28	0	28	24	24	0.66	0.71	6.2	43.3	29.4	74.9
9	49	29	20	11	40	0.69	0.68	5.5	199.2	65.8	415.3
10	22	22	0	0	22	0.67	0.74	6.2	32.5	23.1	50.4
2	5	5	0	0	5	-	-	-	-	-	-
5	1	1	0	0	1	-	-	-	-	-	-

Population number (Pop), population sampling size as total number of individuals samples (N_total_ = all individuals; N_adults_ = adults sampled; N_larvae_ = larvae sampled), and as corrected for family structure with full siblings removed (N_larvae_ = number larval samples retained after removing all but one full sibling from each family group; N_total_ = total sample size after removing all but one full sibling from each family group), and genetic diversity metrics including observed heterozygosity (H_o_) expected heterozygosity (H_e_), allelic richness (AR) corrected for smallest sample size, effective population size estimate (N_e_) using LDNe and a 95% confidence interval of N_e_ (N_e, low_ and N_e, high_) as estimated using a jackknifing approach. “Inf.” indicates infinite upper confidence intervals for N_e_. Note that Populations 2 and 5 were not included in population genetic analyses due to small sample sizes.

### DNA extraction, genotyping, and marker screening

Whole genomic DNA was extracted using DNeasy 96 Blood & Tissue Kit (QIAGEN) at the Molecular Ecology Research Lab at the University of Washington’s School of Aquatic and Fishery Sciences. *Hyla wrightorum* microsatellite markers were developed by the Evolutionary Genetics Core Facility at Cornell University and are described in S1 Table, including GenBank accession numbers. Polymerase chain reaction (PCR) was used to amplify DNA for multiplexed loci using Multiplex PCR kits (QIAGEN). Reactions consisted of 0.2 μM of each primer, 1X Qiagen Multiplex PCR Master Mix, and 1μl DNA in a 10 μl reaction. PCR conditions followed QIAGEN guidelines and included an initial activation step of 15 minutes at 95°C, followed by 25 cycles through three steps: denaturation (30 seconds at 94°C), annealing (90 seconds at 60°C), and extension (90 seconds at 72°C). All PCR reactions were performed on C1000 Touch or S1000 thermal cyclers (Bio-RAD). PCR products were genotyped using 3730*xl* 96-Capillary Genetic Analyzer (Applied Biosystems) at Yale University’s DNA Analysis Facility (New Haven, CT). Genotypes were analyzed using the software GENEMAPPER 4.1 (Applied Biosystems). Individuals with poor-quality genotype data were processed again through PCR and genotyping, and in a few cases DNA extractions were performed again provided sufficient tissue remained. Individuals with > 25% missing data after three genotyping attempts were discarded from downstream analyses.

Alleles were binned using the program TANDEM [23]. Any alleles occurring in < 3 individuals and > 1 repeats from other alleles at the locus were confirmed by re-amplifying and genotyping the samples. Loci were screened for the presence of linkage disequilibrium using the log-likelihood ratio statistic for each pair of loci in each population (GenePop 1.2 [24]). Loci were also screened for deviations from Hardy-Weinberg equilibrium (HWE) using Fisher’s exact test as implemented in GenePop, and the presence of null alleles was evaluated with Micro-Checker [25]. All screening procedures were performed first using adult samples only (three populations) and then again with all population samples (adults and larvae) following full sibling removal (description follows).

Larval samples can bias population genetics findings by artificially inflating genetic differentiation due to family structure [26]; therefore, we screened all larval samples for full siblings using the program COLONY 2.0 [27]. All genotype data (with and without siblings removed) are available via figshare (doi: 10.6084/m9.figshare.3365293). Microsatellite loci were first screened for linkage disequilibrium or deviations from HWE using adult genotypes to identify any loci that may affect detection of siblings. When full siblings were detected, one sibling was retained from each family in the final dataset.

### Genetic diversity and population structure

We calculated genetic diversity estimates of expected heterozygosity (*H*_E_), observed heterozygosity (*H*_O_), allelic richness (AR) rarified to the smallest number of sampled individuals per population, and *F*_*IS*_ [28]. We also calculated two global measures of genetic differentiation, global *F*_*ST*_ [28] and *G'*_ST_ [29]. A hierarchical analysis of molecular variance (AMOVA) among and within sampling locations was performed using ARLEQUIN 3.1 [30] with 10,000 permutations to assess significance. Pairwise genetic distance (between each pair of sample sites) was examined in three ways: *D*_*ps*_, a method of measuring genetic differentiation based on proportion of shared alleles [31], *F*_*ST*_ [28] bootstrapped with 10,000 replicates to determine significant difference from zero, and Slatkin’s linearized *F*_*ST*_, calculated as *F*_*ST*_ /(1- *F*_*ST*_) [32]. Here we report Slatkin’s linearized *F*_*ST*_ as lin*F*_*ST*_. Genetic diversity, global genetic differentiation, and pairwise genetic differentiation metrics were calculated using MSA 4.05 [33].

We estimated effective population size (*N*_*e*_) for each sampling location and for all individuals combined. *N*_*e*_ for each sampling location was estimated using the linkage disequilibrium method (LDNe) of Waples and Do [34] as implemented in NeEstimator V2 [35]. LDNe has been shown to perform well in simulations, even in the presence of migration [36]. However, migration can inflate population-level *N*_*e*_ estimates, and overall *N*_*e*_ estimates can be downwardly biased if sub-structure exists among populations [37]. In order to account for these potential biases, we used a heirarchical estimation of total *N*_*e*_ that accounts for population structure. We estimated total *N*_*e*_ following Wright [21] as implemented for hierarchical estimation of total *N*_*e*_ in Holleley et al. [38]:

$$Total\;N_{e}=\frac{\sum_{i}Local\;N_{e}}{1-F_{ST}}$$

Total *N*_*e*_ was calculated with mean local *N*_*e*_ estimates and upper and lower 95% jackknifed confidence interval values. A value of 10,000 was used in the case of local *N*_*e*_ estimates with infinite upper confidence values [19].

Evidence of recent bottlenecks was evaluated using the program BOTTLENECK 1.2.02 [39]. We performed a Wilcoxon signed rank test (1,000 iterations), which is appropriate for tests evaluating fewer than 20 loci [39], to determine if observed heterozygosity was higher than expected in a population at mutation drift equilibrium, indicating that a genetic bottleneck had occurred. We followed recommended parameters for microsatellite markers (100% of mutations following the infinite allele model and a variance of 0.36 for the geometric distribution of the model) [39], and a Bonferroni correction was applied to adjust the critical p-value for multiple comparisons. We also performed a mode-shift test to determine whether distortion of the expected L-shape frequency distribution was detected and attributable to a recent bottleneck in any populations [40].

To evaluate whether individuals sampled at a location likely originated from the local population, we used an individual assignment analysis as implemented in GeneClass2 [41]. We used the Bayesian approach of Rannala and Mountain [42] with 1,000 Monte Carlo-simulated individuals per sample. The designation of individuals as migrants was based on the likelihood of the individual genotype within the population where the individual was sampled (L_home), which is appropriate in cases where source populations may be missing from the samples (i.e., some populations may not have been sampled) [41]. Individual-based hierarchical population structure was evaluated using the Bayesian clustering program STRUCTURE 2.3.4 [43]. Each sampling site was treated as an independent putative population with a total of *n* putative populations. Ten iterations of each *K* from 1 to *n* + 1 were run for 500,000 cycles with a burn-in of 50,000 cycles. Given the close geographic proximity of these populations and the likelihood that they are relatively closely related, we allowed admixture and correlated allele frequencies. Incorporating sampling information using the LOCPRIOR model has been shown to perform better than models without LOCPRIOR in cases of weak but significant genetic structure [43]. Because we anticipated weak population structure for *H*. *wrightorum* based on previous studies of sympatric pond-breeding species [19, 44], we conducted STRUCTURE analyses with LOCPRIOR. We then compared our results to those obtained without using LOCPRIOR. We determined the most likely *K* using the delta-*K* method [45] in which the most likely value of *K* is assessed by the second-order rate of change in the log-likelihood. A delta-*K* value cannot be calculated for *K* = 1. However, *K* = 1 is assumed most likely for runs in which *K* = 1 has the greatest log-likelihood [46]; α, the Dirichlet parameter for degree of admixture, varies throughout a run rather than converging [47]; and assignment to genetic clusters when *K* > 1 tends to be highly admixed within individuals [47]. We identified terminal clusters (*K* = 1) by first examining log-likelihood and secondarily by visually inspecting α and individual admixture results. This analysis was repeated for genetic clusters in which both *K* > 1 and *n* > 1 to identify hierarchical population structure until terminal clusters were described [45]. Individuals from a given sampling location were not subdivided in hierarchical analyses. Rather, sampling location groups were kept intact to evaluate hierarchical structure across rather than among sampling locations. STRUCTURE output visualizations were constructed using the program DISTRUCT 1.1 [48].

### Landscape genetic analysis

To examine our hypotheses of landscape effects on population genetic structure, we first constructed four landscape connectivity surfaces using CIRCUITSCAPE [49]. CIRCUITSCAPE uses circuit theory to simulate gene flow (i.e., “current”) through a resistance surface in which landscape features hypothesized to promote gene flow are assigned low resistances, and landscape features hypothesized to inhibit gene flow are assigned high resistances. CIRCUITSCAPE allows gene flow across multiple pathways and reports pairwise summations of resistance between sampling locations. Modeling multiple pathways is appropriate for dryland anurans with potentially high dispersal ability [19, 44]. To generate pairwise resistance data, we built raster maps of resistance (low to high) using spatial data describing stream networks, canopy cover, and topography (slope). Four resistance surfaces were created to represent four simple models of landscape effects on gene flow: isolation-by-distance (uniform resistance across the landscape); isolation-by-slope (high resistance for steep regions, and low resistance across flat land), connectivity-by-canopy (low resistance with high canopy cover, high resistance in areas with low or no canopy), and connectivity-by-stream (low resistance along riparian corridors, defined as a 100 m buffer surrounding streams). All spatial data were obtained from publicly available sources and are described in detail in the supporting information, S8 Table. A geographic information system (ArcGIS 10.1, Environmental Systems Research Institute) was used to catalog and manipulate landscape data and generate resistance raster maps. Each resistance map was scaled for hypothesized landscape resistance to gene flow from 1–100 where 1 indicates low resistance and 100 indicates high resistance. Values of slope and percent canopy cover were scaled linearly from 1–100 to create resistance surfaces, and stream resistance surface consisted of values of 1 (low resistance) within stream buffers and 100 (high resistance) outside stream buffers. The scale of resistance values was arbitrary and was designed to reflect hypothesized relationships between landscape features and genetic connectivity. All resistance surfaces were scaled to 30 m resolution—the minimum resolution available for canopy cover (see S8 Table). The spatial extent of resistance rasters ensured a buffer of at least 7 km from the edge of the raster to a given sample location. We selected a 7 km buffer distance because 7 km is the farthest nearest-neighbor distance between any sampling locations. With this grain and extent, we were able to perform all CIRCUITSCAPE analyses in the pairwise source/ground modeling mode and using a cell connection scheme of eight neighbors, allowing maximum freedom of current flow. Finally, we evaluated correlations between pairwise landscape resistances, reported these correlations in the supporting information (S7 Table), and did not include resistances with r > 0.7 in the same models.

We evaluated relationships between landscape resistance and population genetic differentiation (as measured by *D*_*ps*_ and lin*F*_*ST*_) in two ways. First, we first employed a causal modeling framework as implemented in Cushman et al. [50]. By calculating a series of Mantel tests [51] and partial Mantel tests, we assessed the relationship between each landscape resistance matrix and the genetic distance matrix while sequentially partialling out the influence of the other landscape resistance matrices. This approach is effective in identifying spurious correlations between genetic distance and landscape processes, particularly when landscape factors may be correlated [52].

Additionally, we evaluated relationships between pairwise genetic distance and pairwise landscape resistances using a mixed-effects modeling approach [53]. Through mixed-effects modeling, explanatory variables (pairwise landscape resistances) are treated as fixed effects, and sampling locations are treated as random effects in a pairwise covariance structure to account for non-independent values in distance matrices. All resistance variables (fixed effects) were mean-centered, and we evaluated model fit with three assessment criteria. We calculated Akaike’s Information Criterion corrected for small sample sizes (AICc [54–55]) and Bayesian Information Criterion (BIC [56]). We also evaluated models using the R^2^_β_ statistic [57]. R^2^_β_ compares a model with fixed and random effects (pairwise landscape distance or resistance and sampling location) to a null model with only the random effect (sampling location) and an intercept. All analyses were performed in R version 2.14.0 (R Development Core Team, 2012), using a modified version of lme4 [58] and PBKRTEST [59] for R^2^_β_ calculation as described in van Strien et al. [53].

## Results

**Fig 2:**
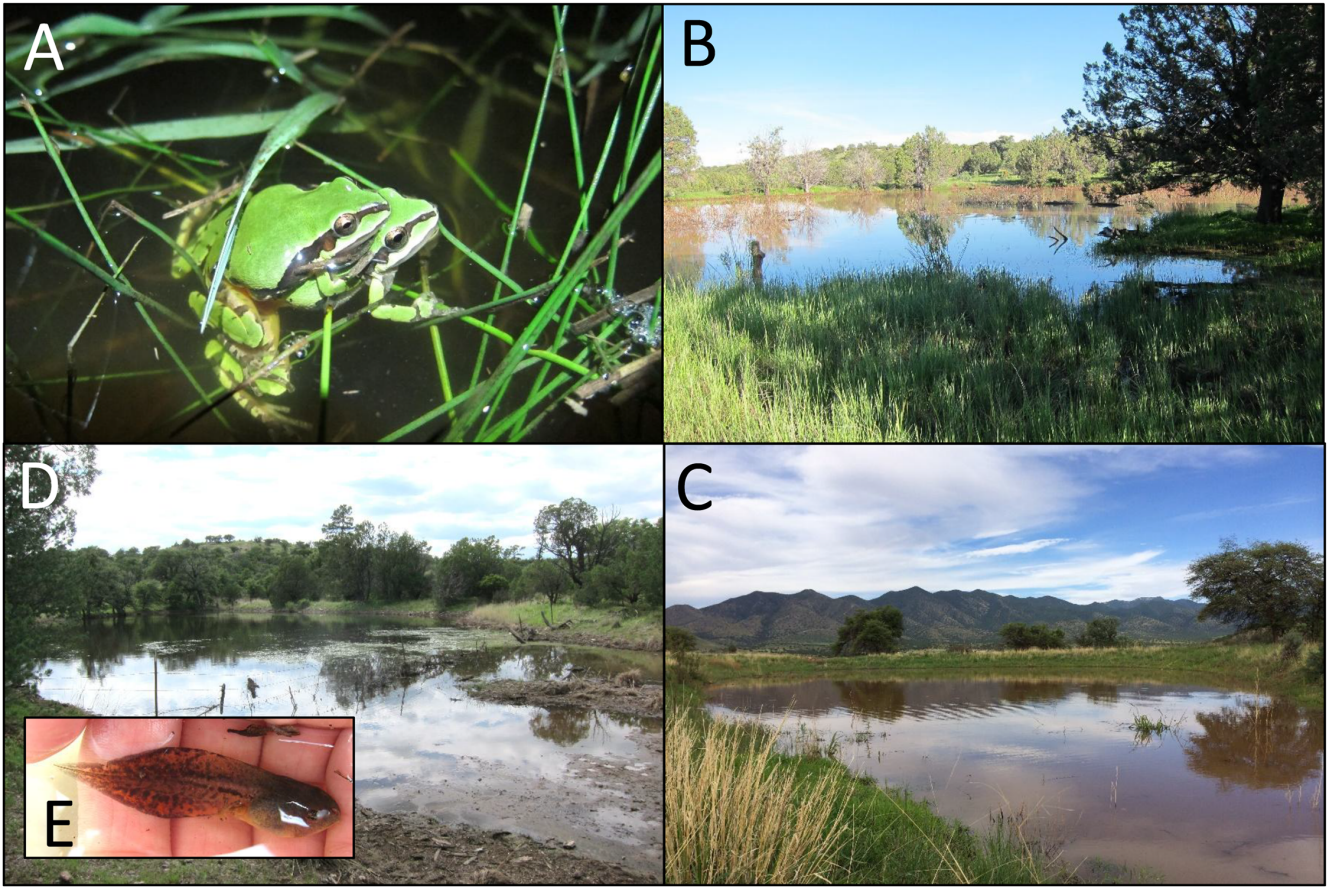
*Hyla wrightorum* individuals and breeding sites. (A) *H*. *wrightorum* breeding pair at Site 1; (B) Site 10; (C) Site 2; (D) Site 4; and (E) *H*. *wrightorum* larvae sampled at Site 4. Sites in B, C, and D are all new localities for this species.

We sampled *H*. *wrightorum* individuals at 10 locations for a total of N = 299 individuals, 82 of which were adults (Figs 1 and 2, Table 1). Four of the 10 sampling locations had no previous record of *H*. *wrightorum* presence (sites 2, 4, 5, and 10). Two locations had insufficient sample sizes for population-level analyses (populations 2 and 5); among the remaining 8 populations, the mean sample size was 36.6 individuals, with a minimum of 19 and a maximum of 50. We retained only 1 full sibling from each family identified, reducing the final sample size to a total of *N* = 221 (Table 2). Among the 8 populations with sufficient sample sizes for population level analyses, mean sample size with full siblings excluded was 27.6, with a minimum of 19 and maximum of 40 (Table 1).

**Table 2:** Results of *Hyla wrightorum* sibling identification among larval samples.

Pop	N_families_	Mean family size sampled	Max N_sibs_
3	30	1.43	7
4	23	2.09	5
6	20	1.70	6
7	37	1.35	3
8	24	1.17	2
9	40	1.23	3

Summary of COLONY results for family structure identification. Sampling location number (Pop) for sites at which larvae were sampled, number of families identified, mean family size, and maximum number of siblings sampled within a family are shown.

All individuals were genotyped for 17 novel polymorphic microsatellite loci (Table 3; microsatellite loci described in S1 Table, including GenBank accession numbers). Genotyping failed for nine larval individuals (not included in total sample size) in which individuals were missing > 25% genotype data despite multiple re-run attempts. In some cases, a second DNA extraction attempt was performed. Some of these larvae were either very small when sampled or were found dead among live larvae and salvaged as voucher specimens. We suspect that species identification was incorrect for these individuals (in the case of very small larvae that may have been *H*. *arenicolor*) or DNA was too degraded for successful genotyping (in the case of salvaged individuals). We found deviations from Hardy-Weinberg expectations at 21 of the 136 sample/locus combinations (15%) prior to application of a Bonferroni correction. However, with a Bonferroni correction applied, we found only 5 instances of significant deviation from HWE (S2 Table). Because these deviations occurred across five different loci and three different populations, we elected to retain all loci in our analyses. Significant LD for two markers pairs were found in only one of 8 populations each, and no evidence of consistent LD was observed for any marker pair (S3 Table). We also found no evidence of null alleles or large allele dropout from Micro-Checker.

**Table 3:** Polymorphic microsatellite loci for *Hyla wrightorum*.

Locus	H_e_	H_o_	Allelic attributes			
			Min	Mean	Max	Richness
Hwri1316	0.742	0.744	157	214.93	237	5.99
Hwri1422	0.762	0.656	188	200.06	212	5.66
Hwri2688	0.677	0.646	236	262.07	272	5.54
Hwri2932	0.627	0.637	200	209.00	224	5.53
Hwri3318	0.783	0.802	250	259.77	274	6.72
Hwri4093	0.634	0.570	145	160.17	169	5.10
Hwri4269	0.452	0.429	152	154.64	172	3.70
Hwri4370	0.829	0.798	177	244.82	269	10.14
Hwri10374	0.722	0.637	321	328.64	341	5.14
Hwri12115	0.860	0.843	186	227.02	270	10.89
Hwri16672	0.719	0.764	177	195.06	265	6.91
Hwri20812	0.818	0.816	296	340.12	376	8.40
Hwri23452	0.620	0.591	155	169.05	175	4.24
Hwri29495	0.778	0.754	257	280.89	305	6.61
Hwri30215	0.695	0.634	303	311.02	327	5.55
Hwri30594	0.719	0.548	233	245.13	265	6.02
Hwri34484	0.650	0.636	135	142.00	163	4.88

Attributes of each of 17 microsatellite loci. Allelic richness was computed using the minimum sample number (smallest population minus missing data) for each locus. Additional information, including attributes by population and results of Hardy-Weinberg equilibrium testing, linkage equilibrium, and null allele screening are included in the supporting information.

**Fig 3:**
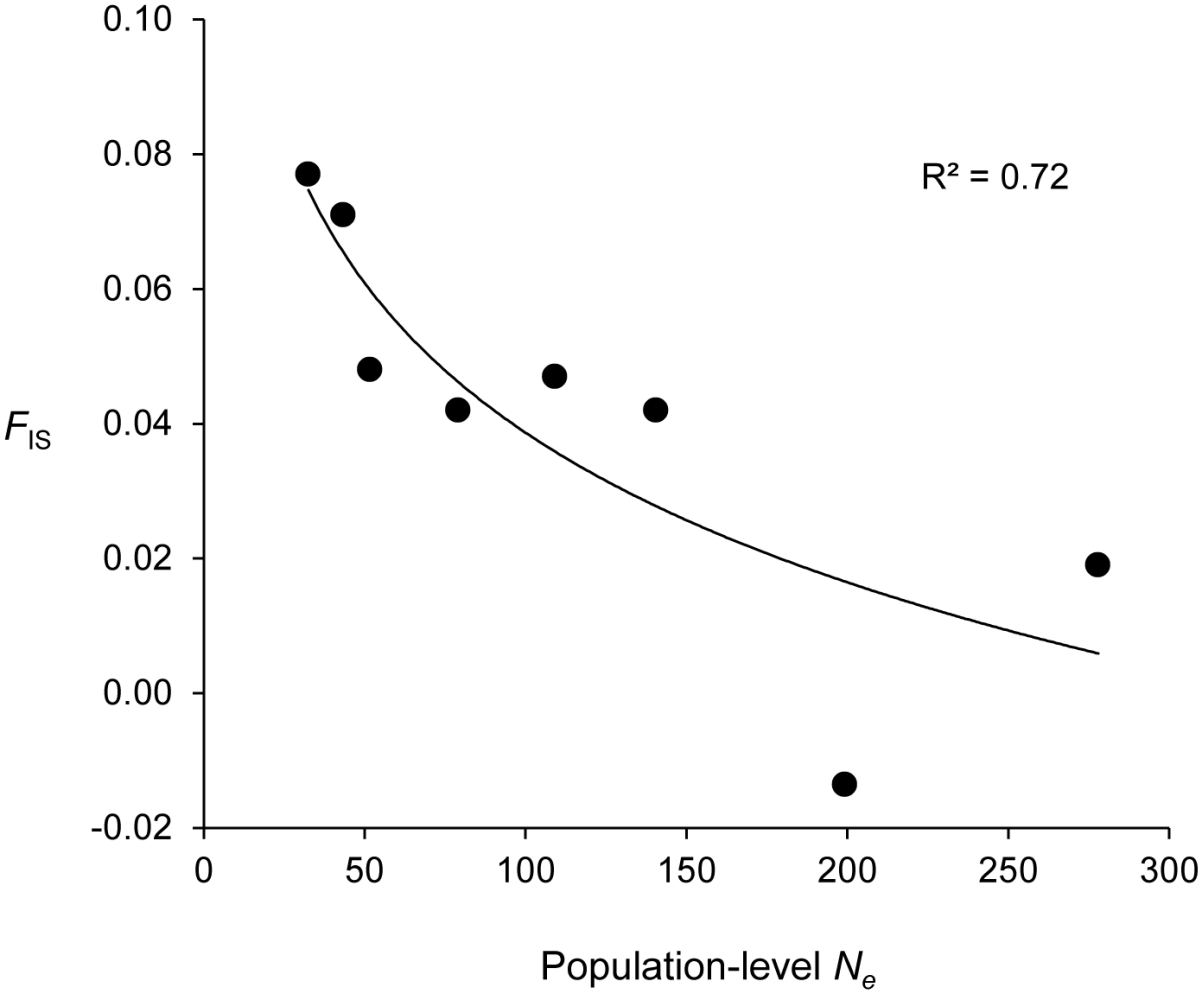
Population-level *N*_*e*_ and *F*_IS_ for *Hyla wrightorum*. Relationship between effective population sizes (*N*_*e*_) derived from LDNe (*N*_e_, x-axis) and *F*_IS_ (y-axis) for 8 *H*. *wrightorum* populations sampled in the Huachuca Mountains and Canelo Hills, Arizona. The black line and R^2^ reflect a logarithmic regression with the equation *y* = 0.03*ln* (*x*) + 0.19. All genetic diversity metrics are derived from 17 microsatellite loci described in the text.

Estimated total *N*_*e*_ was 972.1 (mean), with a jackknifed 95% confidence interval of 401.7 to 30402.4. Population-level *N*_*e*_ averaged 116.7 individuals per population, with a minimum estimate of 32.5 and a maximum estimate of 277.9. Confidence intervals for *N*_*e*_ ranged from 23.1 to infinite values (Table 1). Observed heterozygosity averaged across all loci ranged from 0.647 (Site 4) to 0.695 (Site 7), and expected heterozygosity averaged across all loci ranged from 0.671 (Site 4) to 0.736 (Site 7). Allelic richness ranged from 5.47 (Site 4) to 6.24 (Site 8). Information for population and locus pairs, including observed heterozygosity, expected heterozygosity, allelic attributes, and *F*_*IS*_ are included in the supporting information, S2 Table. We observed a negative relationship between *N*_*e*_ and *F*_*IS*_ (Fig 3, R^2^ = 0.72), which may indicate some inbreeding in populations with smaller effective population sizes. Wilcoxon tests for bottlenecks found evidence of a recent bottleneck among individuals at only one location (Site 7), and mode-shift tests found no evidence of bottlenecks (Table 4).

**Table 4:** Bottleneck test results for *Hyla wrightorum*.

Pop	IAM	Mode-shift
1	0.274	-
3	0.463	-
4	0.322	-
6	0.066	-
7	**0.003**	-
8	0.537	-
9	0.189	-
10	0.112	-

Results of the two bottleneck tests: Wilcoxon signed rank test with 100% mutations under the infinite allele model and a variance of the geometric distribution = 0.36 (IAM) and a mode-shift test. P-values are shown for the IAM test, and results significant after a Bonferroni correction (critical p-value = 0.006) are shown in bold. Mode-shift test results with a dash (-) indicate a normal L-shaped mode. Results are based on 17 microsatellites and 8 populations for *H*. *wrightorum*.

Global genetic differentiation measures provided evidence for genetic structure among populations with values of *F*_*ST*_ = 0.04 and *G’*_*ST*_ = 0.19. A hierarchical AMOVA revealed modest but significant genetic structure with 4.2% variation explained among populations and 3.4% explained among individuals (supporting information, S4 Table). Pairwise *F*_*ST*_ revealed significant differentiation among populations; 25 of 28 pairwise comparisons were significantly different from zero (*F*_*ST*_, Bonferroni correction applied, Table 5). The three non-significant pairwise comparisons all involved Site 6, one of the most central sampling sites (Fig 1). Site 6 was not significantly different from Sites 1, 7 or 8.

**Table 5:** *F*_*ST*_, lin*F*_*ST*_, *D*_*ps*_ values for *Hyla wrightorum* populations.

*F*_*ST*_	1	3	4	6	7	8	9	10
1		0.0342	0.0245	**0.0136**	0.0247	0.0396	0.0621	0.0382
3	0.0001		0.0348	0.0357	0.0464	0.0585	0.0891	0.0883
4	0.0007	0.0001		0.0218	0.0362	0.0527	0.0670	0.0749
6	**0.0112**	0.0001	0.0003		**0.0086**	**0.0151**	0.0345	0.0292
7	0.0001	0.0001	0.0001	**0.0297**		0.0212	0.0291	0.0382
8	0.0001	0.0001	0.0001	**0.0051**	0.0001		0.0301	0.0390
9	0.0001	0.0001	0.0001	0.0001	0.0001	0.0001		0.0444
10	0.0001	0.0001	0.0001	0.0001	0.0001	0.0001	0.0001	
Lin*F*_*ST*_								
	1	3	4	6	7	8	9	10
3	0.035							
4	0.025	0.036						
6	0.014	0.037	0.022					
7	0.025	0.049	0.038	0.009				
8	0.041	0.062	0.056	0.015	0.022			
9	0.066	0.098	0.072	0.036	0.030	0.031		
10	0.040	0.097	0.081	0.030	0.040	0.041	0.046	
*D*_*ps*_								
	1	3	4	6	7	8	9	10
3	0.278							
4	0.256	0.275						
6	0.237	0.286	0.249					
7	0.249	0.293	0.277	0.212				
8	0.302	0.315	0.311	0.244	0.240			
9	0.333	0.380	0.330	0.269	0.246	0.253		
10	0.309	0.392	0.370	0.277	0.303	0.307	0.283	

Pairwise *F*_*ST*_ values (top, upper diagonal) with p-values (lower diagonal) obtained after 10,000 permutations. Population numbers are included at top of columns and left of rows, and *F*_*ST*_ values found non-significant after a Bonferroni correction (critical p-value = 0.002) are shown in bold. Linearized *F*_*ST*_ (Lin*F*_*ST*_) and proportion of shared alleles (*D*_*ps*_) are also shown (middle and bottom of table).

**Fig 4:**
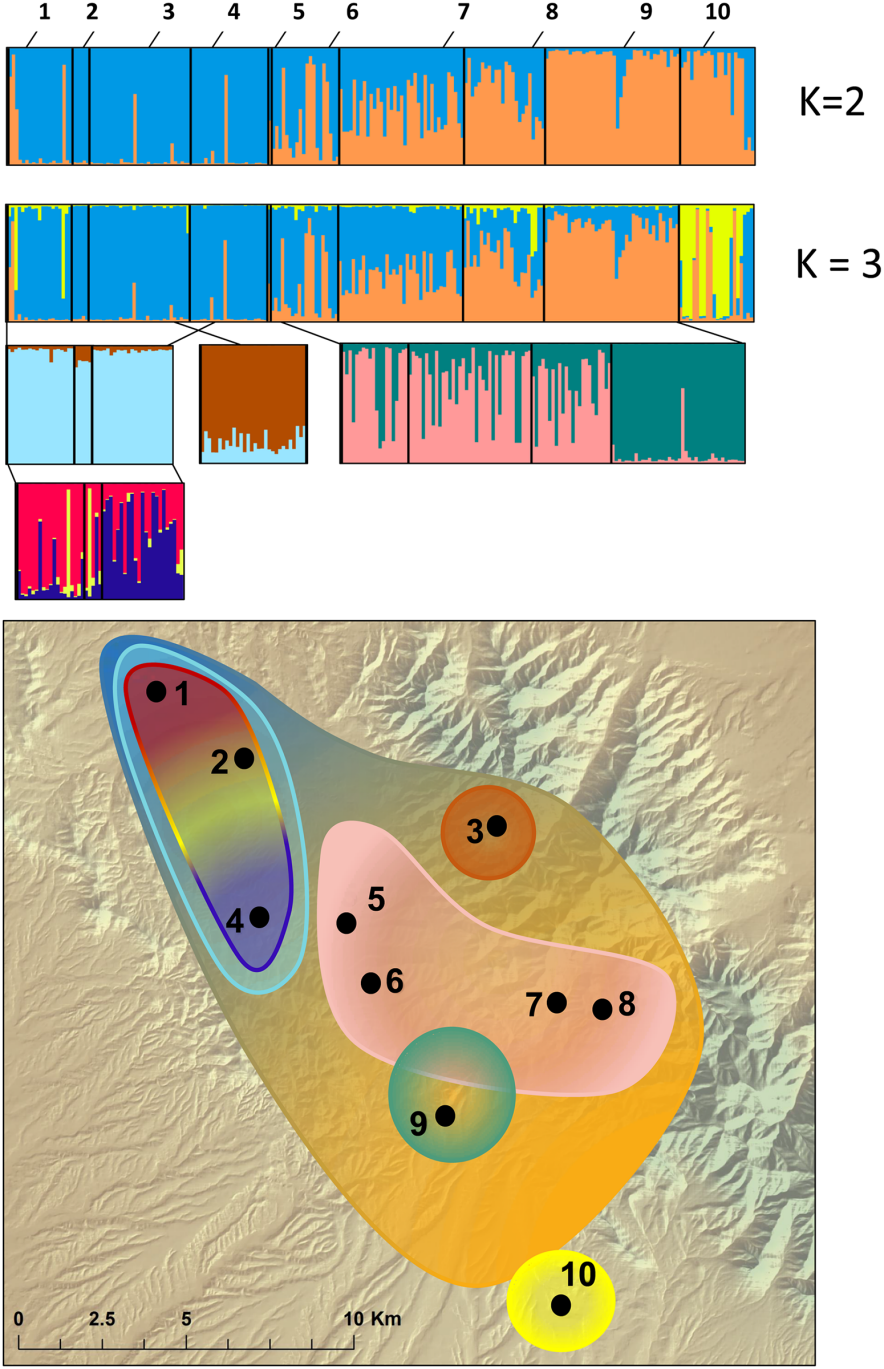
Structure results for *Hyla wrightorum*. Numbers correspond to sampling locations (Fig 1, Table 1). Each vertical bar represents one individual. Colors indicate the most likely genetic cluster assignments. Black vertical bars denote individuals from the same sampling locations. Each cluster was hierarchically analyzed for nested structure; nested structure results are shown directly below the original cluster. Hierarchical analyses were repeated until terminal clusters (*K* = 1) were reached. Note that results for *K* = 2 (upper-most Structure plot) and *K* = 3 (just below *K* = 2) clusters are shown for all individuals, because both had similar support (S6 Table). Hierarchical analyses are shown following *K* = 3 for all individuals. Nested, colored outlines on the map correspond to population clusters.

Despite significant genetic differentiation between populations, 50.2% of individuals were assigned to populations other than their sampling location (Table 6, S5 Table). In addition, 5 individuals had low probabilities (<5%) of assignment to all reference populations, indicating these individuals may be recent migrants from unsampled populations. Admixture between populations was also evident in the Structure analyses, which provided support for modest hierarchical clustering among *H*. *wrightorum* individuals with evidence of isolation-by-distance (Fig 4). Support for *K* = 2 and *K* = 3 clusters across all individuals was similar (S6 Table). For both *K* = 2 and *K* = 3, we found support for a general shift from one group (blue, the dominant group for Sites 1, 2, 3, and 4) to a second group (orange, dominant for Sites 9 and 10). This pattern followed a north to south trend, with individuals from centrally located sites assigned to both clusters (Fig 4). In the *K* = 3 scenario, we identified a third genetic group within Site 10. Fig 4 shows both *K* = 2 and *K* = 3 for all individuals, and hierarchical results are shown for the *K* = 3 scenario. Site division for hierarchical analysis included sites 1, 2, 3, and 4, which had few individuals assigned to the second group (orange), and sites 6, 7, 8, and 9, which had a modest to dominant presence of the second group. Hierarchical results for the alternate *K* = 2 scenario are included in the supporting information, with the only difference from the *K* = 3 scenario being the inclusion of site 10 in the second group (S6 Table). At the secondary level (within major clusters identified across all individuals), we found support for separate genetic clusters in Sites 3 and 9. Sites 5, 6, 7 and 8 were ultimately grouped together with strongest support for K = 1. We found support for *K* = 3 clusters for the group encompassing Sites 1, 2, and 4; however, visual inspection of structure within this group does not support strong differentiation between populations but rather a possible isolation-by-distance pattern. STRUCTURE analyses without LOCPRIOR largely agree with the results of the analyses performed with LOCPRIOR (supporting information, S7 Table). There was only one discrepancy between analyses with and without LOCPRIOR: analyses with LOCPRIOR found modest support for genetic clusters among sites 1, 2, 3, and 4, whereas analyses without LOCPRIOR suggest that those sites may be panmictic. Delta-*K* tables for all species and genetic clusters are included in the supporting information (S6 and S7 Tables).

**Table 6:** GeneClass2 results for *H*. *wrightorum*.

		Assigned population									
Pops	*N*	1	3	4	6	7	8	9	10	Other	Total
1	19	10	1	2	2	1		1	2		9
3	30	5	22			2				1	8
4	23	6	2	8	3	1	2		1		15
6	20	3	1	1	8	2	3	2			12
7	37	2			4	18	6	3	3	1	19
8	24	1	3		3	1	13	1		2	16
9	40	2	1		2	7	12	13	3		27
10	22	3					1	3	14	1	8

GeneClass2 results for genetic assignment of *H*. *wrightorum* individuals. Table includes sample location (Pop), sample size (*N*), assigned population number, and total individuals assigned to locations other than their sampling location (Total). "Other" indicates low (<5%) probability of assignment to any reference population. Underlined values represent individuals assigned to their sampling location. Empty cells indicate value of zero.

We found multiple lines of evidence that, of the spatial attributes examined, distance is a primary driver of population structure. We also found evidence that slope and canopy may affect population structure. Of the four Mantel tests examining hypothesized relationships between spatial and landscape variables (Distance, Slope, Canopy, and Stream) and genetic distance (*D*_*ps*_ and lin*F*_*ST*_), only Distance was significantly univariately related to *D*_*ps*_ (Mantel r = 0.50, p-value = 0.015). Lin*F*_*ST*_ was not significantly univariately related to any landscape variables (Table 7). Distance remained significantly related to *D*_*ps*_ in three partial Mantel tests controlling for Slope, Canopy, and Stream, and Distance was significantly related to lin*F*_*ST*_ in two of three partial Mantel tests (controlling for Slope and Canopy). These findings support the expected linear relationship between resistance (of a uniform landscape) and genetic distance in an isolation-by-distance scenario [49] (S1 Fig). For both measure of genetic distance, isolation-by-slope had support as a driver of genetic structure after accounting for Canopy, and there was also a significant effect of canopy after accounting for slope (Table 7; but note the high correlation between canopy and distance, S7 Table). There was no support for stream as a driver of genetic distance in this dataset.

**Table 7:** Mantel and partial Mantel results for *Hyla wrightorum*.

	Distance		Slope		Canopy		Stream	
*D*_*ps*_	r	p-value	r	p-value	r	p-value	r	p-value
Mantel	**0.50**	**0.02**	0.34	0.12	0.21	0.25	0.17	0.29
*partial Mantel*, *controlled for*:								
Distance	-	-	0.42	0.08	0.30	0.84	0.15	0.31
Slope	**0.55**	**0.01**	-	-	**0.62**	**0.01**	0.17	0.33
Canopy	**0.53**	**0.01**	**0.65**	**< 0.01**	-	-	0.17	0.30
Stream	**0.50**	**0.03**	0.38	0.14	0.21	0.26	-	-
**Lin*F***_***ST***_								
Mantel	0.36	0.09	0.39	0.11	0.08	0.40	0.09	0.38
*partial Mantel*, *controlled for*:								
Distance	-	-	0.43	0.08	0.31	0.82	0.07	0.41
Slope	**0.41**	**0.06**	-	-	**0.49**	**0.04**	0.09	0.39
Canopy	**0.45**	**0.02**	**0.59**	**0.01**	-	-	0.08	0.41
Stream	0.35	0.11	0.38	0.12	0.08	0.41	-	-

Results for Mantel and partial Mantel tests in a causal modeling framework to examine relationships between the genetic distance matrices of *D*_*ps*_ (proportion shared alleles, top portion of table) and lin*F*_*ST*_ (bottom portion of table) with four spatial distance matrices derived from the resistance surfaces representing isolation-by-distance, isolation-by-slope, connectivity-by-canopy, and connectivity-by-stream for *H*. *wrightorum*. Mantel r and associated p-values are shown for each analysis.

Mixed-effects models also support distance as the primary driver of genetic structure for this dataset (Table 8). The information criteria used (AICc and BIC) were consistent in indicating that the best model was the model with Distance only for both *D*_*ps*_ and *linF*_*ST*_ (Table 8). The standard threshold for identifying the best models in a suite of candidate models is a difference of 2 [51], and the ΔAICc and ΔBIC values for the next best model of isolation-by-slope were well above this threshold for both measures of genetic distance. Distance had the highest R^2^_*β*_ value among univariate models for *D*_*ps*_, and Distance was tied with Slope for the highest R^2^_*β*_ value for lin*F*_*ST*_. Of the more complex models, those that included Canopy outperformed those with Distance for mixed-effects models according to the R^2^_*β*_ metric (Table 8), but not according to AICc and BIC.

**Table 8:** Mixed-effects models and landscape genetic results for *Hyla wrightorum*.

*D*_*ps*_	R^2^_β_	BIC	ΔBIC	AICc	ΔAICc
Isolation-by-distance (Distance)	0.28	-89.4	0	-93	0
Isolation-by-slope (Slope)	0.22	-78.9	10.5	-82.5	10.5
Connectivity-by-canopy (Canopy)	0.18	-72.4	17	-76	17
Connectivity-by-stream (Stream)	0.17	-72	17.4	-75.6	17.4
Distance + Slope	0.51	-78.2	11.2	-82.1	10.9
Canopy + Slope	0.55	-69.6	19.9	-73.5	19.5
Stream + Slope	0.47	-64.1	25.3	-68	25
Distance + Slope + Stream	0.60	-62.3	27.1	-66.3	26.7
Canopy + Slope + Stream	0.63	-53.5	35.9	-57.5	35.5
**Lin*F***_***ST***_					
Isolation-by-distance (Distance)	0.22	-121.3	0	-124.9	0
Isolation-by-slope (Slope)	0.22	-113.5	7.8	-117.1	7.8
Connectivity-by-canopy (Canopy)	0.1	-106.1	15.2	-109.7	15.2
Connectivity-by-stream (Stream)	0.12	-106.2	15.1	-109.7	15.2
Distance + Slope	0.43	-109.2	12.1	-113.1	11.8
Canopy + Slope	0.46	-100.1	21.2	-104.1	20.8
Stream + Slope	0.36	-96.9	24.4	-100.8	24.1
Distance + Slope + Stream	0.52	-92	29.3	-96	28.9
Canopy + Slope + Stream	0.54	-82.8	38.5	-86.8	38.1

Results of mixed-effects models for evaluating relationships between distance matrices of *D*_*ps*_ (proportion shared alleles, top portion of table) and lin*F*_*ST*_ (bottom portion of table) and landscape resistances. Spatial data are described in full in the supporting information (S8 Table). Canopy and Distance were not included in the same models due to high collinearity (see S9 Table for correlations between distance matrices and S10 Table for resistance values). All R^2^_β_ correlation coefficients were positive.

## Discussion

We found a higher number of breeding sites than previously described and evidence of larger than expected population sizes for this isolated group of populations of *H*. *wrightorum* within the HMCH region. We found support for significant genetic differentiation between many populations despite evidence of admixture. The influence of spatial and landscape variables on population structure was evident, with significant support for isolation-by-distance and some support for the effects of slope and canopy on genetic structure. Taken together, these results suggest that populations of *H*. *wrightorum* in the Huachuca Mountains may constitute a metapopulation in which the spatial and temporal variability of available breeding habitat may contribute to local extinctions, colonizations, and exchange of individuals. Similar evidence exists for many pond breeding amphibians [60].

The discovery of previously unknown breeding sites for this species as part of this research effort supports two alternative hypotheses for why this species has not been observed at some historical sites over the last few decades. First, previous survey efforts may simply have missed active breeding sites due to spatial or temporal mismatches between survey efforts and breeding activity. Alternatively, natural population fluctuations or metapopulation dynamics may account for the absence of this species at historical breeding sites and the discovery of the species at new sites over the last two decades. Over the course of an intensive 3-week field season in 2014, we found *H*. *wrightorum* in 4 new locations (Sites 2, 4, 5, and 10). Site 4 is a confirmed breeding site (larvae present), and Site 10 is a presumed breeding site (> 20 adults sampled in a breeding chorus). Site 10 extends the known range of contiguous breeding sites in the Huachuca Mountains southeast by roughly 7 km. Additionally, limited observations of *H*. *wrightorum* have been reported at a small number of wetland habitats at Rancho Los Fresnos, Sonora, Mexico, south of the Huachuca Mountains [6]. The Los Fresnos population is presumed to be small and is typically described as an “outlier” from the Huachuca and Canelo Hills populations due to its isolation and distance from other known breeding sites in the Huachuca Mountains. However, the newly discovered Site 10 is approximately intermediate in distance between Los Fresnos and previously described breeding sites, representing a significant reduction in the spatial gap between these sites. Survey efforts for this species in 2015 (after the conclusion of sampling efforts for this study) confirmed *H*. *wrightorum* at three additional breeding locations, all previously undescribed (Fig 1; CSG, K. Strickler unpublished data). Two 2015 sites are also intermediate to previously known sites and the Los Fresnos location. In general, the discovery of new sites during this study supports the hypotheses that these populations exist within a metapopulation in the region, and that more intensive survey efforts are required for detection of this species. Results from the GeneClass analysis revealed that some individuals may have originated from one or more unsampled populations in the HMCH region.

In consideration of these observations, continued survey efforts exploring additional plausible breeding sites within the region are recommended. For example, within a 7 km buffered area encompassing all sites in this study, over 90 ponds with intermediate hydroperiods were identified using satellite imagery (publically available imagery, accessible via Google Earth). Many of these ponds were not visited in 2014 due to logistical and time constraints, and many have not been surveyed for this species. Temporal dynamics such as the frequency, timing, and duration of the period during which these ponds are wet versus dry is not known. Future research might aim to quantify the hydrologic dynamics of these ponds, and how they may change in the future, in order to help managers better understand the spatiotemporal variability of available breeding habitat. Additionally, *H*. *wrightorum* are thought to prefer or require emergent vegetation in breeding habitat [7], and they likely avoid ponds with predatory or competitive species present [61]. These two criteria alone may exclude many of the intermittent ponds within 7 km of known breeding sites; however, specific habitat associations and requirements of *H*. *wrightorum* remain uncertain. Additional survey efforts aimed at detecting this species and refining knowledge of breeding habitat will be critical in identifying other potential breeding sites for this species within the HMCH region and for optimal management of known breeding sites [62].

Significant pairwise *F*_*ST*_ comparisons, AMOVA results, and evidence of hierarchical structure among populations indicate considerable genetic differentiation between populations, with a significant influence of spatial and/or landscape variables. Individual-based cluster analyses of *H*. *wrightorum* most strongly supported a predominant pattern of isolation-by-distance. Additional hierarchical analyses reveal some genetic clusters strongly associated with a certain locality (Sites 3, 9, and 10). However, the presence of heterogeneity in assignment of individuals to clusters (Sites 9 and 10) or the lack of support from analyses that did not incorporate sampling location information (Site 3) indicates weak clustering at these locations. Admixture between sites was also supported with the assignment test results. In cases of more complete genetic isolation, we might expect to see near-perfect correlation of cluster assignments with sampling location, as observed in the one of the region’s aquatic macroinvertebrates, *Abedus herberti*, which requires perennial aquatic habitat [63].

Our findings suggest significant genetic structure of *H*. *wrightorum* populations in the HMCH region, which is in contrast to some sympatric pond-breeding anurans, including *Anaxyrus cognatus*, *Scaphiopus couchii*, and *Spea multiplicata*, found to have panmictic population structure across similar spatial scales [19, 44]. However, the pond-breeding anurans in those studies are more desiccation tolerant, more mobile, have higher fecundity and larger population sizes, and are able to better utilize more ephemeral breeding ponds due to short larval periods (i.e., one- to three-week larval periods for species with the lowest larval requirements versus one to a few months for hylids in the region) [64–65]. These life history differences may explain the greater genetic differentiation observed for *H*. *wrightorum* in this study. By contrast, Storfer et al. [20] report much greater genetic differentiation among populations of an endangered pond breeding salamander, *Ambystoma mavortium stebbinsi*, in the San Rafael Valley and foothills of the HMCH region. They found no support for isolation-by-distance among populations and instead found evidence that genetic drift and small population sizes are responsible for high levels of genetic differentiation among populations. We found that *H*. *wrightorum* population structure was most similar to *H*. *arenicolor* in the Huachuca Mountains [19], a predominantly stream-dwelling amphibian and *H*. *wrightorum*’s only congener in the region.

Our analyses comparing the relationship between genetic structure and landscape variables of distance, slope, canopy cover, and streams provide additional support for the role of spatial and landscape factors in the genetic structure we observed. Taken together, we found strong support for physical distance as a driver of genetic distance. We also found some support for the role of slope and canopy cover, indicating that populations that are closer together are more highly related, and that flatter, more forested areas may play a role in facilitating gene flow. Correlation between canopy cover and distance highlights the difficulty of interpreting collinear landscape distances [52]. However, our results provide strong support for distance as the most likely factor affecting genetic structure. Although we did not detect an influence of streams or a strong influence of canopy cover in this study, the study region receives considerable precipitation during most summers and has high canopy cover throughout this portion of the species range. If favorable landscapes (i.e., those that promote gene flow) occur at a high density on the landscape, detecting their influence can be difficult using genetic approaches [66]. Additionally, we used simple linear or binary resistance values for the landscape variables in this study, and it is possible that genetic distance is related to landscape variables in non-linear ways. Finally, we hypothesized and tested three landscape variables we expected might influence genetic structure of *H*. *wrightorum* populations, but additional landscape variables not considered in this study may also explain some portion of *H*. *wrightorum* genetic structure. Thus, these findings do not rule out the possibly important role of landscape variables other than distance in connecting populations.

Total and population-level *N*_*e*_ estimates had a high degree of uncertainty as evidenced by wide confidence intervals. This uncertainty may be due in part to migration and other demographic complexities that complicate the estimation of *N*_*e*_, particularly for metapopulations. Although LDNe is considered one of the most reliable methods for estimating *N*_*e*_ with a single sample in time [36], migration and population structure can bias estimates. Migration can upwardly bias *N*_*e*_ due to immigrants creating a larger total pool of parents than a sample with only local breeders [37]. Given the high degree of gene flow observed between populations, migration may have inflated LDNe estimates within populations. By contrast, samples with sub-structure may downwardly bias estimates of *N*_*e*_ calculated with LDNe due to linkage disequilibrium created by the mixture of multiple gene pools [37]. We attempted to avoid this bias by implementing the hierarchical calculation of Wright [21] which accounts for population structure and has been shown to outperform non-hierarchical total *N*_*e*_ estimates [38]. However, migration and population dynamics likely make characterizing “true” *N*_*e*_ values difficult regardless of the method employed, and the *N*_*e*_ estimates in this study should be interpreted with caution.

Despite uncertainty in our *N*_*e*_ estimates, our results suggest that previous assumptions of consistently small population sizes, with *N*_*c*_ estimates near or below 30 individuals [6], likely underestimate population sizes of this species. For highly fecund species or r-selected species, such as amphibians, differential recruitment may result in a *N*_*e*_ / *N*_*c*_ ratio that is quite low [67]. Thus, it is likely that *N*_*c*_ of *H*. *wrightorum* populations in the study region are larger than the estimated *N*_*e*_ values reported here. Still, concern regarding the size of *H*. *wrightorum* populations in the region is warranted. We found small mean family sizes among larval samples, indicating that variance in reproductive success may be modest in this species. Thus, the ratio of *N*_*e*_ / *N*_*c*_ may be closer to 1 than for other amphibians. Mean *N*_*e*_ was less than 100 for the overall estimate and for half the populations we sampled, and the 95% confidence interval of *N*_*e*_ included values < 100 for all populations, as is typical for many pond breeding amphibians [68]. *N*_*e*_ < 100 may indicate these populations are at risk of genetic depletion due to genetic drift and/or inbreeding [3], particularly if gene flow is sufficiently low. In this case, the relationship between *N*_*e*_ and *F*_*IS*_ indicates that inbreeding may occur in some populations.

Despite genetic connectivity between populations and evidence of higher than expected *N*_*e*_, the dynamics of this system and small number of known populations indicate that these populations are of conservation concern. *N*_*e*_ values reported here should be considered in the context of temporal uncertainty, particularly because environmental instability, demography, and other factors that vary through time can influence population dynamics [69]. Although the breeding habitat of *H*. *wrightorum* in the HMCH region occurs in a largely remote area with little human activity or development, many of the breeding ponds are frequented by cattle. The effects of grazing and other disturbances associated with livestock are currently not known for this amphibian. Predation by the invasive American bullfrog (*Lithobates catesbeianus*) could significantly reduce the number of adults at breeding ponds—particularly if choruses are small [13]. Site 7, the only *H*. *wrightorum* population with any evidence of a bottleneck, may have faced decades of predation from bullfrogs that established at a perennial pond just meters away. An intensive bullfrog eradication program began in the area in the early 2000’s [14], and it is possible that historical bullfrog predation at Site 7 may have contributed to or driven the bottleneck we observed (T. Jones, personal communication). *Batrachochytrium dendrobatidis* (Bd), a disease associated with the decline and/or extinction of many amphibians worldwide [70], is documented in the region [71] and is known to infect *H*. *wrightorum* [72]. *Hyla wrightorum* from northern Arizona showed no increase in mortality risk when exposed to Bd in a controlled, experimental setting [73], but the effects of Bd populations in the HMCH region are not currently known.

On a regional scale, climate change will likely bring drier conditions to the southwestern United States [16] with the possibility of spatial and temporal reduction in aquatic breeding habitat as well as changes in disease dynamics [74]. Shifts in climate along with other human modifications to the landscape will also likely result in larger and more intense wildfires in the region [15]. Such fires are often accompanied by major flooding and erosion events that can scour or fill breeding sites with sediment. We suggest continued survey efforts to determine occupancy of breeding habitats by *H*. *wrightorum*, and we suggest additional research aimed at quantifying both the habitat requirements of and threats to this species to characterize suitable breeding habitat. Such efforts will help inform efficient and effective management of intermittent ponds to promote the persistence and continued connectivity of *H*. *wrightorum* populations within the HMCH region.

## Supporting Information

S1 Fig Relationship between genetic distance and uniform landscape resistance for *H*. *wrightorum*. (DOCX)

S1 Table Primer sequences and GenBank Accession numbers for 17 *H*. *wrightorum* microsatellite loci. (DOCX)

S2 Table Microsatellite loci information for each population of *H*. *wrightorum* sampled. (DOCX)

S3 Table Results for test for linkage disequilibrium for *H*. *wrightorum* microsatellite loci. (DOCX)

S4 Table Results for an analysis of molecular variance (AMOVA) for *H*. *wrightorum*. (DOCX)

S5 Table GeneClass2 individual assignment probabilities for *H*. *wrightorum*. (DOCX)

S6 Table Delta K calculations and log likelihoods for Structure output for *H*. *wrightorum* with LOCPRIOR. (DOCX)

S7 Table Delta K calculations and log likelihoods for Structure output for *H*. *wrightorum* without LOCPRIOR. (DOCX)

S8 Table Sources, descriptions, and details for spatial data used in landscape genetic analyses for *H*. *wrightorum*. (DOCX)

S9 Table Pearson correlations coefficiens between all distance matrices (genetic and spatial). (DOCX)

S10 Table Landscape resistance matrices generated using CIRCUITSCAPE. (DOCX)
