# Supplementary material for: Genetic Differentiation, Isolation-by-Distance, and Metapopulation Dynamics of the Arizona Treefrog (Hyla wrightorum) in an Isolated Portion of Its Range
Source: PLoS One. 2016 Aug 9;11(8):e0160655. doi: 10.1371/journal.pone.0160655 (PMC4978385; doi:10.1371/journal.pone.0160655)

**Fig S1. Relationship between genetic distance and uniform landscape resistance for *H. wrightorum*.** Relationships between genetic distance (A: Linearized *F_ST_* ; and B: *D_ps_*) and resistance on a uniform landscape, or null resistance, for pairwise comparisons between 8 *H. wrightorum* populations sampled in the Huachuca Mountains and Canelo Hills, Arizona. The black lines and R^2^ values reflect linear regressions, where A: slope = 0.08 with *t*_26_ = 1.95, p-value = 0.06; and B: slope = 0.22, t_26_ = 2.96, p-value = 0.007. Both genetic diversity metrics are derived from 17 microsatellite loci described in the text.


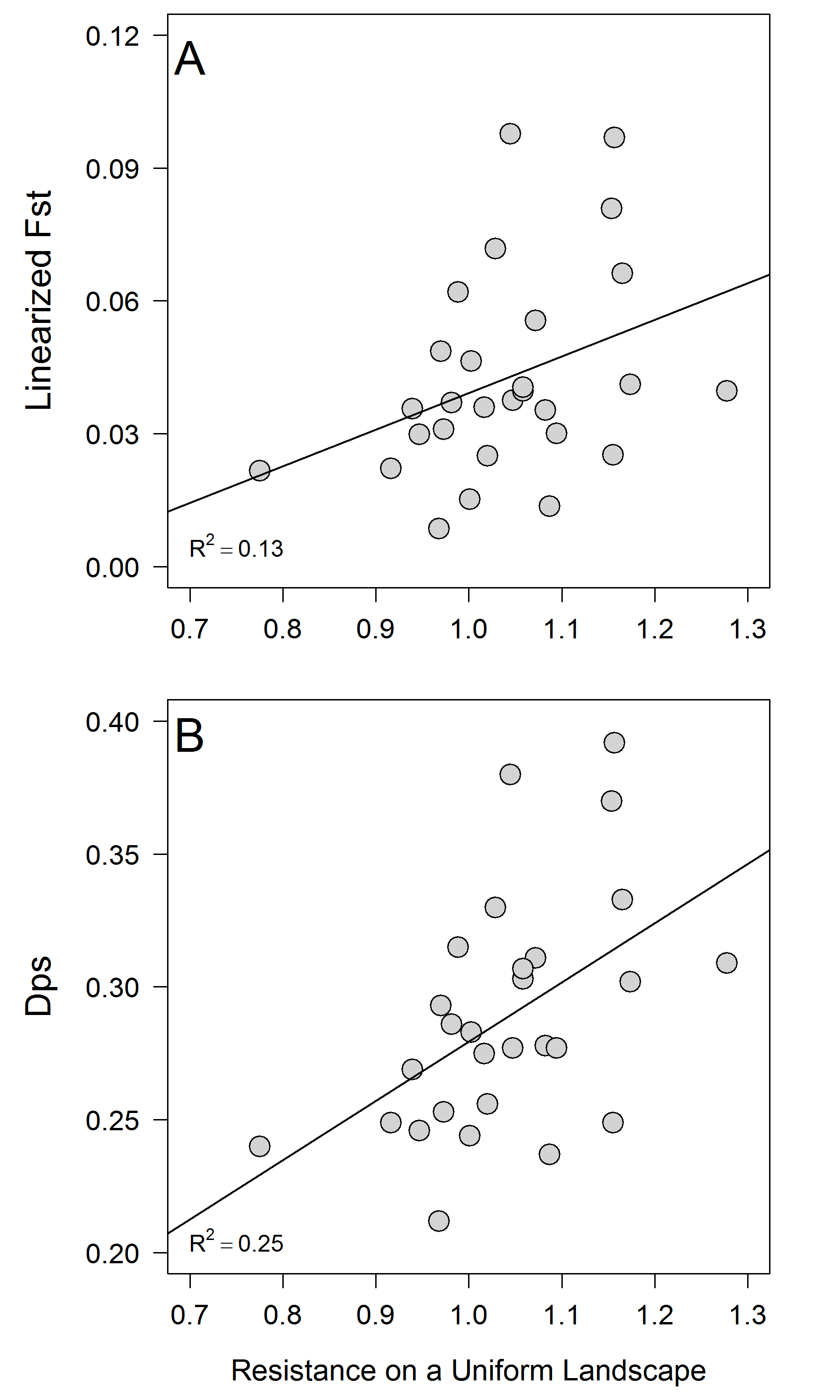

Supplement: S1 Fig — (DOCX) [file pone.0160655.s001.docx]
