# Supplementary material for: Genetic Differentiation, Isolation-by-Distance, and Metapopulation Dynamics of the Arizona Treefrog (Hyla wrightorum) in an Isolated Portion of Its Range
Source: PLoS One. 2016 Aug 9;11(8):e0160655. doi: 10.1371/journal.pone.0160655 (PMC4978385; doi:10.1371/journal.pone.0160655)
Supplement: S1 Table — (DOCX) [file pone.0160655.s002.docx]

| S1 Table. Locus, repeat sequence, GenBank Accession number, and primer sequences for all 17 polymorphic microsatellite loci for *Hyla wrightorum.* Italicized sequences at the 5’ end of the reverse primers are added to promote adenylation of the dye-labeled strand. | | | | |
| --- | --- | --- | --- | --- |
| Locus | Repeat | GenBank | Forward primer (5' - 3') | Reverse primer (5' - 3') |
| Hwri1316 | (CTAT)_14_ | KX086286 | GTACGTGTGACCCTACCCTC | *GTTTCTT*ACCTATAGCTTGCACCACCG |
| Hwri1422 | (ATCT)_13_ | KX086287 | TGTTATGGGCCTCTGACCAG | *GTTTCTT*ACATGTGAAGTGTGCTGCTG |
| Hwri2688 | (CTAT)_11_AT(CTAT)_2_ | KX086288 | GATGTTTGCACGCTTGTCAC | *GTTTCTT*TTGTGCAATTGTTGGCTGAC |
| Hwri2932 | (CTAT)_12_ | KX086289 | GACTGGCTTCCGTGGATTTC | *GTTTCTT*CTGTAAACTCCTGTGCTAGCC |
| Hwri3318 | (TATC)_11_ | KX086290 | AAGATAGGGCCATTCGACCG | *GTTTCTT*ATGGTGCTGTGGAGAGATCC |
| Hwri4093 | (TATC)_13_ | KX086291 | CCAGACAAACCTCAGCCAAC | *GTTTCTT*TGCTTCACATATACTGGAGTGC |
| Hwri4269 | (GACA)_11_(GATA)_7_ | KX086292 | AGCGTGTGGGATTGAATGAC | *GTTTCTT*CTCAAAGACCAGGGAGTGTC |
| Hwri4370 | (TCTG)_10_(TCTA)_10_ | KX086293 | TCCCTCACCCTGGATTGTG | *GTTTCTT*GGGAGGAAACCATGCTTGTG |
| Hwri10374 | (TATC)_9_TAA(TATC)_3_ | KX086294 | GGAGGGTGCAAACGAGAAATG | *GTTTCTT*GCGAGGGATTTGTTGGTTGG |
| Hwri12115 | (CTAT)_14_ | KX086295 | GAACTACTCAACTCCAGCAGC | *GTTTCTT*TACTGTGTCAAGTCCTGCCC |
| Hwri16672 | (TATC)_10_ | KX086296 | ACCTGCTGCTTGGATATTTGC | *GTTTCTT*AGTGTGCTGCTGTATCCTTC |
| Hwri20812 | (TCTA)_12_ | KX086297 | TGTTCTGCTAATGTCCTTCTGC | *GTTTCTT*GCGGCCCTGGATAGACATG |
| Hwri23452 | (ACAT)_10_ | KX086298 | AGCACTGCAGAATATGTTGGTG | *GTTTCTT*GTGCCATACTCTCATTTGTGTG |
| Hwri29495 | (TATC)_12_ | KX086299 | AGTGCTAAGAGGCCTGACAG | *GTTTCTT*TGGAGTGCTGCAGTTCAC |
| Hwri30215 | (TCTA)_16_ | KX086300 | TTTGGGTTTCACTTTGCTTGG | *GTTTCTT*ATGAGGGCATCTTGTGTTGG |
| Hwri30594 | (TCTA)_8_128(TATC)_13_CATC(CA)_7_ | KX086301 | GATGTGTGCAGCAGGTCAC | *GTTTCTT*CTGCACACTTGTCTGTTCCC |
| Hwri34484 | (TACA)_13_ | KX086302 | TGAGGGAACGAATTAAAGGGAC | *GTTTCTT*TGTACTGAAGGCTGGAAGGG |
