# Supplementary material for: Genetic Differentiation, Isolation-by-Distance, and Metapopulation Dynamics of the Arizona Treefrog (Hyla wrightorum) in an Isolated Portion of Its Range
Source: PLoS One. 2016 Aug 9;11(8):e0160655. doi: 10.1371/journal.pone.0160655 (PMC4978385; doi:10.1371/journal.pone.0160655)
Supplement: S2 Table — (DOCX) [file pone.0160655.s003.docx]

| S2 Table. *H. wrightorum* microsatellite loci information by population. Sample size (N) by locus and population (Pop), observed heterozygosity (H_o_), expected heterozygosity (H_e_), minimum allele size (Min A), mean allele size (Mean A), maximum allele size (Max A), number of alleles (A Count), allelic richness (A Rich), *F_IS_*, and p-values of Hardy Weinberg tests (HWE, p-val), where significant HWE tests after a Bonferroni correction (critical p-val = 0.006) are shown in bold. | | | | | | | | | | | |
| --- | --- | --- | --- | --- | --- | --- | --- | --- | --- | --- | --- |
| Locus | Pop | N | *Ho* | *He* | Min A | Mean A | Max A | A Count | A Rich | *F_IS_* | HWE, p-val |
| 1316 | 1 | 19 | 0.895 | 0.812 | 201 | 219.53 | 233 | 5 | 5.00 | -0.12 | 0.394 |
| 1316 | 3 | 29 | 0.931 | 0.815 | 157 | 220.52 | 237 | 8 | 7.19 | -0.15 | 0.251 |
| 1316 | 4 | 23 | 0.696 | 0.724 | 201 | 210.57 | 233 | 5 | 5.00 | 0.03 | 0.658 |
| 1316 | 6 | 20 | 0.650 | 0.722 | 201 | 214.60 | 233 | 4 | 4.00 | 0.09 | 0.711 |
| 1316 | 7 | 37 | 0.649 | 0.703 | 201 | 214.73 | 233 | 5 | 4.89 | 0.07 | 0.385 |
| 1316 | 8 | 24 | 0.708 | 0.716 | 169 | 211.42 | 237 | 8 | 7.70 | 0.00 | 0.020 |
| 1316 | 9 | 40 | 0.650 | 0.637 | 201 | 213.20 | 233 | 5 | 4.91 | -0.03 | 0.533 |
| 1316 | 10 | 22 | 0.773 | 0.804 | 201 | 214.91 | 233 | 6 | 5.86 | 0.03 | 0.157 |
| 1422 | 1 | 19 | 0.632 | 0.745 | 188 | 200.42 | 212 | 6 | 6.00 | 0.14 | 0.209 |
| 1422 | 3 | 30 | 0.667 | 0.762 | 188 | 201.80 | 208 | 5 | 4.95 | 0.12 | 0.224 |
| 1422 | 4 | 23 | 0.609 | 0.702 | 188 | 198.78 | 212 | 6 | 5.95 | 0.13 | 0.438 |
| 1422 | 6 | 20 | 0.550 | 0.787 | 188 | 198.00 | 212 | 6 | 5.95 | 0.29 | 0.016 |
| 1422 | 7 | 37 | 0.649 | 0.780 | 188 | 200.76 | 212 | 6 | 5.40 | 0.16 | **0.003** |
| 1422 | 8 | 24 | 0.708 | 0.740 | 188 | 201.33 | 212 | 6 | 5.75 | 0.03 | 0.821 |
| 1422 | 9 | 40 | 0.750 | 0.742 | 188 | 198.65 | 208 | 5 | 4.86 | -0.02 | 0.816 |
| 1422 | 10 | 22 | 0.682 | 0.837 | 188 | 200.73 | 212 | 6 | 6.00 | 0.18 | 0.472 |
| 2688 | 1 | 19 | 0.632 | 0.714 | 244 | 261.58 | 268 | 5 | 5.00 | 0.10 | 0.458 |
| 2688 | 3 | 30 | 0.767 | 0.678 | 248 | 262.47 | 268 | 6 | 5.14 | -0.14 | 0.037 |
| 2688 | 4 | 23 | 0.739 | 0.660 | 244 | 262.87 | 268 | 5 | 4.82 | -0.13 | 0.786 |
| 2688 | 6 | 20 | 0.600 | 0.708 | 244 | 260.50 | 268 | 5 | 5.00 | 0.14 | 0.010 |
| 2688 | 7 | 37 | 0.595 | 0.662 | 252 | 263.57 | 272 | 6 | 5.05 | 0.10 | 0.076 |
| 2688 | 8 | 24 | 0.667 | 0.770 | 236 | 261.08 | 268 | 7 | 6.87 | 0.13 | **0.001** |
| 2688 | 9 | 40 | 0.625 | 0.661 | 252 | 262.60 | 268 | 5 | 4.72 | 0.05 | 0.307 |
| 2688 | 10 | 22 | 0.545 | 0.564 | 244 | 261.91 | 268 | 4 | 4.00 | 0.02 | 0.848 |
| 2932 | 1 | 18 | 0.667 | 0.716 | 200 | 210.22 | 224 | 7 | 7.00 | 0.06 | 0.391 |
| 2932 | 3 | 30 | 0.667 | 0.710 | 204 | 210.60 | 224 | 6 | 5.68 | 0.05 | 0.761 |
| 2932 | 4 | 23 | 0.609 | 0.521 | 204 | 207.22 | 220 | 4 | 4.00 | -0.18 | 1.000 |
| 2932 | 6 | 20 | 0.850 | 0.688 | 200 | 209.50 | 224 | 7 | 6.80 | -0.25 | 0.901 |
| 2932 | 7 | 37 | 0.595 | 0.702 | 204 | 210.70 | 224 | 6 | 5.22 | 0.15 | 0.114 |
| 2932 | 8 | 23 | 0.565 | 0.546 | 204 | 208.17 | 224 | 6 | 5.52 | -0.05 | 0.673 |
| 2932 | 9 | 39 | 0.462 | 0.467 | 204 | 206.62 | 224 | 6 | 5.05 | 0.00 | 0.176 |
| 2932 | 10 | 22 | 0.682 | 0.663 | 204 | 209.00 | 224 | 6 | 5.79 | -0.04 | 0.707 |
| 3318 | 1 | 19 | 0.737 | 0.777 | 250 | 260.32 | 274 | 6 | 6.00 | 0.04 | 0.469 |
| 3318 | 3 | 30 | 0.833 | 0.802 | 250 | 258.80 | 274 | 7 | 6.85 | -0.05 | 0.869 |
| 3318 | 4 | 23 | 0.870 | 0.810 | 250 | 258.26 | 274 | 7 | 6.94 | -0.09 | 0.990 |
| 3318 | 6 | 20 | 0.950 | 0.808 | 250 | 259.60 | 274 | 6 | 6.00 | -0.19 | 0.168 |
| 3318 | 7 | 37 | 0.757 | 0.811 | 250 | 258.00 | 274 | 7 | 6.53 | 0.06 | 0.071 |
| 3318 | 8 | 23 | 0.739 | 0.796 | 250 | 258.61 | 274 | 6 | 5.95 | 0.06 | 0.385 |
| 3318 | 9 | 40 | 0.800 | 0.726 | 250 | 260.05 | 274 | 7 | 6.68 | -0.11 | 0.037 |

| S2 Table, Continued. | | | | | | | | | | | |
| --- | --- | --- | --- | --- | --- | --- | --- | --- | --- | --- | --- |
| Locus | Pop | N | *Ho* | *He* | Min A | Mean A | Max A | A Count | A Rich | *F_IS_* | HWE, p-val |
| 3318 | 10 | 22 | 0.727 | 0.737 | 250 | 264.55 | 274 | 7 | 6.97 | 0.00 | 0.497 |
| 4093 | 1 | 19 | 0.684 | 0.784 | 145 | 158.58 | 165 | 5 | 5.00 | 0.12 | 0.160 |
| 4093 | 3 | 29 | 0.379 | 0.416 | 145 | 162.79 | 165 | 5 | 4.83 | 0.08 | 0.346 |
| 4093 | 4 | 23 | 0.522 | 0.584 | 145 | 161.35 | 169 | 6 | 5.94 | 0.10 | 0.147 |
| 4093 | 6 | 20 | 0.500 | 0.699 | 145 | 160.20 | 165 | 5 | 5.00 | 0.28 | 0.193 |
| 4093 | 7 | 36 | 0.694 | 0.689 | 145 | 159.89 | 165 | 5 | 4.98 | -0.02 | 0.072 |
| 4093 | 8 | 22 | 0.682 | 0.678 | 145 | 160.82 | 165 | 5 | 4.98 | -0.02 | 0.375 |
| 4093 | 9 | 40 | 0.525 | 0.551 | 145 | 161.30 | 165 | 5 | 4.91 | 0.04 | 0.488 |
| 4093 | 10 | 21 | 0.571 | 0.674 | 145 | 156.43 | 165 | 5 | 4.90 | 0.14 | 0.568 |
| 4269 | 1 | 19 | 0.421 | 0.408 | 152 | 154.42 | 172 | 4 | 4.00 | -0.05 | 0.717 |
| 4269 | 3 | 30 | 0.433 | 0.437 | 152 | 155.00 | 164 | 3 | 2.95 | 0.00 | 0.841 |
| 4269 | 4 | 23 | 0.130 | 0.204 | 152 | 152.78 | 164 | 3 | 2.97 | 0.36 | 0.093 |
| 4269 | 6 | 20 | 0.550 | 0.613 | 152 | 156.70 | 172 | 4 | 4.00 | 0.09 | 0.424 |
| 4269 | 7 | 37 | 0.622 | 0.619 | 152 | 156.43 | 172 | 4 | 4.00 | -0.01 | 0.031 |
| 4269 | 8 | 24 | 0.417 | 0.451 | 152 | 153.67 | 164 | 3 | 2.99 | 0.07 | 0.253 |
| 4269 | 9 | 40 | 0.450 | 0.466 | 152 | 154.00 | 164 | 3 | 3.00 | 0.03 | 0.282 |
| 4269 | 10 | 22 | 0.409 | 0.415 | 152 | 154.09 | 164 | 3 | 3.00 | 0.00 | 0.269 |
| 4370 | 1 | 19 | 0.895 | 0.862 | 189 | 245.84 | 269 | 9 | 9.00 | -0.05 | 0.633 |
| 4370 | 3 | 30 | 0.833 | 0.856 | 177 | 243.27 | 265 | 11 | 9.86 | 0.02 | 0.123 |
| 4370 | 4 | 23 | 0.783 | 0.826 | 201 | 249.26 | 269 | 9 | 8.60 | 0.04 | 0.754 |
| 4370 | 6 | 19 | 0.789 | 0.836 | 201 | 248.79 | 269 | 9 | 9.00 | 0.04 | 0.482 |
| 4370 | 7 | 37 | 0.730 | 0.849 | 201 | 245.27 | 269 | 10 | 8.49 | 0.13 | 0.282 |
| 4370 | 8 | 24 | 0.750 | 0.763 | 201 | 247.58 | 269 | 9 | 8.33 | 0.01 | 0.386 |
| 4370 | 9 | 40 | 0.875 | 0.842 | 185 | 240.95 | 265 | 10 | 8.88 | -0.05 | 0.201 |
| 4370 | 10 | 22 | 0.727 | 0.796 | 185 | 237.64 | 265 | 9 | 8.69 | 0.08 | 0.037 |
| 10374 | 1 | 19 | 0.684 | 0.801 | 321 | 329.74 | 341 | 5 | 5.00 | 0.14 | 0.585 |
| 10374 | 3 | 27 | 0.704 | 0.758 | 321 | 330.70 | 341 | 5 | 4.70 | 0.06 | 0.019 |
| 10374 | 4 | 23 | 0.783 | 0.753 | 321 | 329.09 | 341 | 5 | 5.00 | -0.05 | 0.648 |
| 10374 | 6 | 20 | 0.650 | 0.791 | 321 | 330.50 | 341 | 5 | 5.00 | 0.17 | 0.041 |
| 10374 | 7 | 37 | 0.568 | 0.650 | 321 | 328.08 | 341 | 5 | 4.76 | 0.12 | 0.653 |
| 10374 | 8 | 22 | 0.545 | 0.682 | 321 | 327.73 | 341 | 5 | 4.98 | 0.19 | 0.073 |
| 10374 | 9 | 40 | 0.525 | 0.546 | 321 | 324.85 | 341 | 5 | 4.79 | 0.03 | 0.322 |
| 10374 | 10 | 22 | 0.636 | 0.799 | 321 | 328.45 | 341 | 6 | 5.98 | 0.20 | 0.401 |
| 12115 | 1 | 19 | 0.789 | 0.868 | 186 | 228.95 | 250 | 11 | 11.00 | 0.08 | 0.494 |
| 12115 | 3 | 30 | 0.900 | 0.877 | 186 | 232.20 | 270 | 13 | 11.29 | -0.04 | 0.337 |
| 12115 | 4 | 23 | 0.826 | 0.844 | 186 | 222.96 | 250 | 10 | 9.74 | 0.01 | 0.239 |
| 12115 | 6 | 20 | 0.900 | 0.892 | 186 | 219.80 | 250 | 11 | 10.94 | -0.02 | 0.892 |
| 12115 | 7 | 36 | 0.861 | 0.874 | 194 | 230.78 | 250 | 11 | 9.55 | 0.01 | 0.228 |
| 12115 | 8 | 24 | 0.875 | 0.849 | 186 | 223.25 | 250 | 10 | 9.53 | -0.04 | 0.168 |
| 12115 | 9 | 40 | 0.775 | 0.798 | 186 | 228.40 | 254 | 11 | 8.28 | 0.02 | 0.345 |
| 12115 | 10 | 22 | 0.818 | 0.881 | 186 | 229.82 | 254 | 10 | 9.71 | 0.06 | 0.267 |
| 16672 | 1 | 19 | 0.737 | 0.762 | 177 | 194.47 | 217 | 6 | 6.00 | 0.02 | 0.044 |
| 16672 | 3 | 29 | 0.552 | 0.583 | 177 | 186.52 | 265 | 6 | 5.31 | 0.05 | 0.159 |

| S2 Table, Continued. | | | | | | | | | | | |
| --- | --- | --- | --- | --- | --- | --- | --- | --- | --- | --- | --- |
| Locus | Pop | N | *Ho* | *He* | Min A | Mean A | Max A | A Count | A Rich | *F_IS_* | HWE, p-val |
| 16672 | 4 | 23 | 0.826 | 0.668 | 177 | 193.96 | 217 | 4 | 3.97 | -0.25 | 0.288 |
| 16672 | 6 | 20 | 0.850 | 0.721 | 177 | 193.60 | 217 | 6 | 5.90 | -0.20 | 0.751 |
| 16672 | 7 | 37 | 0.865 | 0.703 | 177 | 191.43 | 217 | 7 | 6.02 | -0.24 | 0.116 |
| 16672 | 8 | 24 | 0.708 | 0.790 | 177 | 198.67 | 265 | 9 | 8.32 | 0.09 | 0.619 |
| 16672 | 9 | 40 | 0.850 | 0.756 | 177 | 198.85 | 217 | 6 | 5.20 | -0.13 | 0.577 |
| 16672 | 10 | 22 | 0.727 | 0.765 | 177 | 203.00 | 217 | 7 | 6.85 | 0.04 | 0.106 |
| 20812 | 1 | 19 | 0.842 | 0.788 | 296 | 344.53 | 372 | 9 | 9.00 | -0.08 | 0.940 |
| 20812 | 3 | 29 | 0.759 | 0.736 | 296 | 343.59 | 376 | 8 | 6.92 | -0.04 | 0.620 |
| 20812 | 4 | 23 | 0.826 | 0.836 | 296 | 340.35 | 368 | 8 | 7.65 | 0.00 | 0.770 |
| 20812 | 6 | 20 | 0.900 | 0.842 | 296 | 336.10 | 376 | 8 | 7.90 | -0.08 | 0.743 |
| 20812 | 7 | 37 | 0.784 | 0.805 | 296 | 342.81 | 376 | 9 | 7.79 | 0.02 | 0.539 |
| 20812 | 8 | 23 | 0.870 | 0.853 | 296 | 340.96 | 372 | 7 | 6.97 | -0.03 | 0.971 |
| 20812 | 9 | 40 | 0.775 | 0.828 | 296 | 336.05 | 372 | 7 | 6.90 | 0.06 | 0.263 |
| 20812 | 10 | 22 | 0.773 | 0.857 | 296 | 336.55 | 376 | 9 | 8.73 | 0.09 | 0.766 |
| 23452 | 1 | 19 | 0.474 | 0.542 | 155 | 169.21 | 175 | 3 | 3.00 | 0.12 | 0.446 |
| 23452 | 3 | 27 | 0.769 | 0.668 | 155 | 169.46 | 175 | 5 | 4.97 | -0.16 | 0.016 |
| 23452 | 4 | 23 | 0.348 | 0.590 | 155 | 169.43 | 175 | 4 | 4.00 | 0.41 | **0.004** |
| 23452 | 6 | 20 | 0.650 | 0.606 | 155 | 169.80 | 175 | 4 | 4.00 | -0.09 | 0.392 |
| 23452 | 7 | 37 | 0.622 | 0.631 | 155 | 168.62 | 175 | 3 | 3.00 | 0.01 | 0.791 |
| 23452 | 8 | 24 | 0.500 | 0.568 | 155 | 170.58 | 175 | 4 | 3.95 | 0.11 | 0.832 |
| 23452 | 9 | 40 | 0.775 | 0.638 | 155 | 168.55 | 175 | 4 | 3.73 | -0.22 | 0.322 |
| 23452 | 10 | 22 | 0.591 | 0.719 | 155 | 166.73 | 175 | 5 | 4.85 | 0.17 | 0.027 |
| 29495 | 1 | 19 | 0.737 | 0.797 | 257 | 279.00 | 301 | 6 | 6.00 | 0.06 | 0.644 |
| 29495 | 3 | 30 | 0.700 | 0.797 | 257 | 281.87 | 301 | 5 | 5.00 | 0.11 | 0.184 |
| 29495 | 4 | 23 | 0.696 | 0.719 | 257 | 277.52 | 301 | 5 | 5.00 | 0.02 | 0.722 |
| 29495 | 6 | 20 | 0.650 | 0.718 | 257 | 275.60 | 305 | 6 | 5.95 | 0.08 | 0.245 |
| 29495 | 7 | 37 | 0.784 | 0.755 | 257 | 278.30 | 305 | 7 | 6.22 | -0.05 | 0.218 |
| 29495 | 8 | 24 | 0.750 | 0.837 | 257 | 286.75 | 305 | 7 | 6.95 | 0.10 | 0.237 |
| 29495 | 9 | 40 | 0.900 | 0.783 | 257 | 281.25 | 305 | 7 | 6.40 | -0.16 | 0.867 |
| 29495 | 10 | 22 | 0.818 | 0.815 | 257 | 286.82 | 305 | 7 | 6.73 | -0.02 | 0.114 |
| 30215 | 1 | 19 | 0.632 | 0.653 | 303 | 310.79 | 327 | 5 | 5.00 | 0.02 | 0.713 |
| 30215 | 3 | 29 | 0.414 | 0.561 | 303 | 307.48 | 323 | 4 | 3.88 | 0.26 | 0.195 |
| 30215 | 4 | 23 | 0.565 | 0.574 | 303 | 307.43 | 323 | 5 | 4.48 | 0.00 | 0.711 |
| 30215 | 6 | 20 | 0.650 | 0.683 | 303 | 308.60 | 327 | 6 | 5.90 | 0.04 | 0.022 |
| 30215 | 7 | 37 | 0.649 | 0.714 | 303 | 310.78 | 327 | 5 | 4.90 | 0.09 | 0.395 |
| 30215 | 8 | 24 | 0.708 | 0.779 | 303 | 312.25 | 327 | 6 | 5.95 | 0.08 | 0.640 |
| 30215 | 9 | 40 | 0.725 | 0.770 | 303 | 315.20 | 327 | 6 | 5.47 | 0.05 | 0.472 |
| 30215 | 10 | 22 | 0.727 | 0.822 | 303 | 315.64 | 327 | 6 | 5.86 | 0.11 | 0.232 |
| 30594 | 1 | 17 | 0.529 | 0.656 | 233 | 245.47 | 265 | 7 | 7.00 | 0.18 | 0.383 |
| 30594 | 3 | 30 | 0.367 | 0.527 | 233 | 238.80 | 261 | 5 | 4.82 | 0.30 | 0.035 |
| 30594 | 4 | 23 | 0.348 | 0.672 | 233 | 242.04 | 261 | 5 | 4.93 | 0.48 | **0.004** |
| 30594 | 6 | 20 | 0.500 | 0.777 | 233 | 245.20 | 261 | 5 | 5.00 | 0.35 | 0.038 |
| 30594 | 7 | 36 | 0.611 | 0.804 | 233 | 246.39 | 265 | 6 | 5.95 | 0.24 | 0.046 |

| S2 Table, Continued. | | | | | | | | | | | |
| --- | --- | --- | --- | --- | --- | --- | --- | --- | --- | --- | --- |
| Locus | Pop | N | *Ho* | *He* | Min A | Mean A | Max A | A Count | A Rich | *F_IS_* | HWE,  p-val |
| 30594 | 8 | 24 | 0.708 | 0.764 | 233 | 248.00 | 265 | 7 | 6.55 | 0.06 | 0.406 |
| 30594 | 9 | 40 | 0.700 | 0.810 | 233 | 244.40 | 261 | 6 | 5.42 | 0.13 | 0.394 |
| 30594 | 10 | 21 | 0.619 | 0.744 | 233 | 250.71 | 265 | 7 | 6.40 | 0.16 | 0.151 |
| 34484 | 1 | 19 | 0.737 | 0.704 | 135 | 142.37 | 163 | 5 | 5.00 | -0.06 | 0.365 |
| 34484 | 3 | 28 | 0.750 | 0.666 | 135 | 146.07 | 163 | 5 | 4.58 | -0.14 | 0.304 |
| 34484 | 4 | 23 | 0.826 | 0.729 | 135 | 144.30 | 163 | 4 | 4.00 | -0.15 | 0.756 |
| 34484 | 6 | 20 | 0.550 | 0.601 | 135 | 139.50 | 163 | 5 | 5.00 | 0.07 | 0.368 |
| 34484 | 7 | 37 | 0.784 | 0.760 | 135 | 143.81 | 163 | 5 | 5.00 | -0.04 | 0.928 |
| 34484 | 8 | 22 | 0.273 | 0.458 | 135 | 138.64 | 163 | 5 | 4.85 | 0.40 | **0.001** |
| 34484 | 9 | 40 | 0.575 | 0.620 | 135 | 139.75 | 163 | 5 | 4.85 | 0.07 | 0.200 |
| 34484 | 10 | 22 | 0.591 | 0.661 | 135 | 141.55 | 163 | 5 | 5.00 | 0.10 | 0.397 |
