# Supplementary material for: Genetic Differentiation, Isolation-by-Distance, and Metapopulation Dynamics of the Arizona Treefrog (Hyla wrightorum) in an Isolated Portion of Its Range
Source: PLoS One. 2016 Aug 9;11(8):e0160655. doi: 10.1371/journal.pone.0160655 (PMC4978385; doi:10.1371/journal.pone.0160655)
Supplement: S3 Table — (DOCX) [file pone.0160655.s004.docx]

| S3 Table. Results of test for linkage disequilibrium using the log likelihood ratio statistic as implemented in GenePop. Each pairwise test between markers had 16 degrees of freedom. P-values significant with a Bonferroni correction (critical p-value = 0.003) applied are shown in bold. Note that the only two significant tests were driven by an estimated p-value of zero in one of 8 populations (population-specific results shown for those two marker pairs following overall pairwise comparisons). | | | |
| --- | --- | --- | --- |
| Locus 1 | Locus 2 | Chi^2^ | p-value |
| 1316 | 2688 | 9.46 | 0.893 |
| 1316 | 4093 | 7.07 | 0.972 |
| 1316 | 4370 | 16.02 | 0.452 |
| 1316 | 10374 | 16.98 | 0.387 |
| 1316 | 12115 | 15.43 | 0.494 |
| 1316 | 16672 | 5.26 | 0.994 |
| 1316 | 20812 | 15.21 | 0.509 |
| 1316 | 23452 | 6.85 | 0.976 |
| 1316 | 30594 | 14.13 | 0.589 |
| 1316 | 34484 | 8.99 | 0.914 |
| 1422 | 1316 | 14.97 | 0.526 |
| 1422 | 2688 | 26.43 | 0.048 |
| 1422 | 2932 | 14.47 | 0.564 |
| 1422 | 3318 | 19.21 | 0.258 |
| 1422 | 4093 | 17.30 | 0.367 |
| 1422 | 4269 | 12.67 | 0.697 |
| 1422 | 4370 | 16.02 | 0.452 |
| 1422 | 10374 | 13.14 | 0.662 |
| 1422 | 12115 | 15.28 | 0.505 |
| 1422 | 16672 | 9.34 | 0.899 |
| 1422 | 20812 | 15.09 | 0.518 |
| 1422 | 23452 | 18.91 | 0.274 |
| 1422 | 29495 | 15.49 | 0.489 |
| 1422 | 30215 | 21.50 | 0.160 |
| 1422 | 30594 | 6.07 | 0.987 |
| 1422 | 34484 | 10.94 | 0.813 |
| 2688 | 4093 | 16.69 | 0.406 |
| 2688 | 4370 | 27.08 | 0.041 |
| 2688 | 10374 | 13.64 | 0.626 |
| 2688 | 12115 | 13.37 | 0.645 |
| 2688 | 20812 | 16.90 | 0.392 |
| 2688 | 23452 | 21.06 | 0.176 |
| 2688 | 30594 | 17.67 | 0.344 |
| 2688 | 34484 | 8.30 | 0.940 |
| 2932 | 1316 | 15.28 | 0.504 |
| 2932 | 2688 | 7.91 | 0.951 |
| 2932 | 3318 | 9.56 | 0.888 |
| 2932 | 4093 | 13.43 | 0.641 |
| 2932 | 4269 | 11.57 | 0.773 |

| S3 Table, continued. | | | |
| --- | --- | --- | --- |
| Locus 1 | Locus 2 | Chi^2^ | p-value |
| 2932 | 10374 | 11.55 | 0.774 |
| 2932 | 12115 | 9.33 | 0.899 |
| 2932 | 16672 | 11.09 | 0.804 |
| 2932 | 20812 | 13.33 | 0.648 |
| 2932 | 23452 | 24.23 | 0.085 |
| 2932 | 29495 | 14.16 | 0.587 |
| 2932 | 30215 | 8.08 | 0.946 |
| 2932 | 30594 | 15.56 | 0.484 |
| 2932 | 34484 | 9.38 | 0.897 |
| 3318 | 1316 | 7.18 | 0.970 |
| 3318 | 2688 | 26.03 | 0.054 |
| 3318 | 4093 | 23.17 | 0.109 |
| 3318 | 4269 | 12.48 | 0.710 |
| 3318 | 4370 | 13.70 | 0.621 |
| 3318 | 10374 | 18.29 | 0.307 |
| 3318 | 12115 | 14.55 | 0.558 |
| 3318 | 16672 | 11.88 | 0.753 |
| 3318 | 20812 | 10.62 | 0.832 |
| 3318 | 23452 | 15.35 | 0.499 |
| 3318 | 30594 | 10.02 | 0.866 |
| 3318 | 34484 | 11.49 | 0.778 |
| 4093 | 4370 | 20.98 | 0.179 |
| 4093 | 10374 | 4.60 | 0.997 |
| 4093 | 12115 | 10.63 | 0.832 |
| 4093 | 20812 | 8.36 | 0.937 |
| 4093 | 23452 | 11.46 | 0.780 |
| 4093 | 30594 | 11.38 | 0.786 |
| 4269 | 1316 | 20.59 | 0.195 |
| 4269 | 2688 | 19.59 | 0.239 |
| 4269 | 4093 | 13.82 | 0.612 |
| 4269 | 4370 | 14.95 | 0.528 |
| 4269 | 10374 | 11.54 | 0.775 |
| 4269 | 12115 | 12.27 | 0.725 |
| 4269 | 16672 | 12.72 | 0.693 |
| 4269 | 20812 | 17.44 | 0.358 |
| 4269 | 23452 | 17.04 | 0.383 |
| 4269 | 30594 | 9.85 | 0.874 |
| 4269 | 34484 | 13.61 | 0.628 |
| 10374 | 4370 | 14.14 | 0.589 |
| 10374 | 12115 | 5.54 | 0.992 |
| 10374 | 20812 | 6.22 | 0.986 |
| 10374 | 23452 | 5.54 | 0.992 |
| 10374 | 30594 | 17.57 | 0.350 |
| 12115 | 4370 | 10.42 | 0.844 |
| 12115 | 20812 | 12.19 | 0.731 |

| S3 Table, continued. | | | |
| --- | --- | --- | --- |
| Locus 1 | Locus 2 | Chi^2^ | p-value |
| 16672 | 4093 | 13.32 | 0.649 |
| 16672 | 4370 | 17.78 | 0.337 |
| 16672 | 10374 | 8.68 | 0.926 |
| 16672 | 12115 | 7.40 | 0.965 |
| 16672 | 20812 | 11.13 | 0.802 |
| 16672 | 23452 | 23.44 | 0.102 |
| 16672 | 30594 | 13.57 | 0.631 |
| 16672 | 34484 | 8.67 | 0.926 |
| 20812 | 4370 | 8.24 | 0.941 |
| 20812 | 23452 | 16.88 | 0.393 |
| **20812** | **30594** | **Infinity** | **Highly** |
| 23452 | 4370 | 12.05 | 0.741 |
| 23452 | 30594 | 15.30 | 0.503 |
| 29495 | 1316 | 9.90 | 0.872 |
| 29495 | 2688 | 15.80 | 0.467 |
| 29495 | 3318 | 13.46 | 0.639 |
| 29495 | 4093 | 19.49 | 0.244 |
| 29495 | 4269 | 16.46 | 0.421 |
| 29495 | 4370 | 15.61 | 0.481 |
| 29495 | 10374 | 23.62 | 0.098 |
| 29495 | 12115 | 6.60 | 0.980 |
| 29495 | 16672 | 19.70 | 0.234 |
| 29495 | 20812 | 16.57 | 0.414 |
| 29495 | 23452 | 14.04 | 0.596 |
| 29495 | 30215 | 11.23 | 0.795 |
| 29495 | 30594 | 10.27 | 0.852 |
| **29495** | **34484** | **Infinity** | **Highly** |
| 30215 | 1316 | 20.49 | 0.199 |
| 30215 | 2688 | 14.69 | 0.548 |
| 30215 | 3318 | 12.31 | 0.722 |
| 30215 | 4093 | 19.27 | 0.255 |
| 30215 | 4269 | 19.81 | 0.229 |
| 30215 | 4370 | 8.07 | 0.947 |
| 30215 | 10374 | 11.80 | 0.758 |
| 30215 | 12115 | 12.34 | 0.720 |
| 30215 | 16672 | 25.34 | 0.064 |
| 30215 | 20812 | 13.05 | 0.669 |
| 30215 | 23452 | 16.03 | 0.451 |
| 30215 | 30594 | 26.25 | 0.051 |
| 30215 | 34484 | 14.17 | 0.586 |
| 30594 | 4370 | 8.80 | 0.922 |
| 34484 | 4093 | 13.15 | 0.662 |
| 34484 | 4370 | 15.90 | 0.460 |
| 34484 | 10374 | 6.52 | 0.981 |
| 34484 | 12115 | 13.51 | 0.635 |

| S3 Table, continued. | |  |  |
| --- | --- | --- | --- |
| Locus 1 | Locus 2 | Chi^2^ | p-value |
| Comparison of Loci 20812 and 30594 by population: | | |  |
| Pop | Locus 1 | Locus 2 | P-Value |
| 1 | 20812 | 30594 | 0.643 |
| 3 | 20812 | 30594 | 0.015 |
| 4 | 20812 | 30594 | 0.114 |
| 6 | 20812 | 30594 | 0.338 |
| 7 | 20812 | 30594 | 0.014 |
| 8 | 20812 | 30594 | 0.036 |
| **9** | **20812** | **30594** | **0.000** |
| 10 | 20812 | 30594 | 1.000 |
| Comparison of loci 29495 and 34484 by population: | | |  |
| Pop | Locus 1 | Locus 2 | P-Value |
| 1 | 29495 | 34484 | 1.000 |
| 3 | 29495 | 34484 | 0.121 |
| 4 | 29495 | 34484 | 0.357 |
| 6 | 29495 | 34484 | 0.190 |
| 7 | 29495 | 34484 | 0.082 |
| 8 | 29495 | 34484 | 0.794 |
| **9** | **29495** | **34484** | **0.000** |
| 10 | 29495 | 34484 | 0.325 |
