# Supplementary material for: Genetic Differentiation, Isolation-by-Distance, and Metapopulation Dynamics of the Arizona Treefrog (Hyla wrightorum) in an Isolated Portion of Its Range
Source: PLoS One. 2016 Aug 9;11(8):e0160655. doi: 10.1371/journal.pone.0160655 (PMC4978385; doi:10.1371/journal.pone.0160655)
Supplement: S4 Table — (DOCX) [file pone.0160655.s005.docx]

| S4 Table. AMOVA of 17 microsatellite loci for 8 populations of H. wrightorum in the Huachuca Mountains, AZ. | | | | | | |
| --- | --- | --- | --- | --- | --- | --- |
| Source | d.f. | Sum of squares | Variance components | % Variation | p-value | |
| Among populations | 7 | 139.0 | 0.26 | 4.2 | <0.001 | |
| Among individuals | 207 | 1270.7 | 0.21 | 3.4 | 0.002 | |
| Within individuals | 215 | 1229.0 | 5.76 | 92.4 | <0.001 | |
