# Supplementary material for: Genetic Differentiation, Isolation-by-Distance, and Metapopulation Dynamics of the Arizona Treefrog (Hyla wrightorum) in an Isolated Portion of Its Range
Source: PLoS One. 2016 Aug 9;11(8):e0160655. doi: 10.1371/journal.pone.0160655 (PMC4978385; doi:10.1371/journal.pone.0160655)
Supplement: S5 Table — (DOCX) [file pone.0160655.s006.docx]

| S5 Table. GeneClass2 individual assignment probabilities, calculated using the Bayesian approach of Rannala and Mountain (1997). Sampling location is shown in the left column (Pop), and assignment probabilities for each reference population are shown in the remaining columns. | | | | | | | | |
| --- | --- | --- | --- | --- | --- | --- | --- | --- |
| Pop | 1 | 3 | 4 | 6 | 7 | 8 | 9 | 10 |
| 1 | 0.01 | 0 | 0 | 0.04 | 0 | 0.05 | 0 | 0.13 |
| 1 | 0.02 | 0.01 | 0 | 0.05 | 0.01 | 0.06 | 0 | 0.69 |
| 1 | 0.03 | 0.04 | 0 | 0.14 | 0.11 | 0.30 | 0.66 | 0.07 |
| 1 | 0.07 | 0.42 | 0.02 | 0.07 | 0.08 | 0.14 | 0 | 0.02 |
| 1 | 0.08 | 0.03 | 0.04 | 0.02 | 0.01 | 0.02 | 0 | 0.06 |
| 1 | 0.12 | 0.01 | 0.01 | 0.20 | 0.01 | 0.04 | 0 | 0.04 |
| 1 | 0.19 | 0.19 | 0.08 | 0.34 | 0.30 | 0.13 | 0.03 | 0.20 |
| 1 | 0.21 | 0.01 | 0 | 0.02 | 0.03 | 0.04 | 0 | 0.04 |
| 1 | 0.28 | 0.08 | 0.01 | 0.04 | 0.01 | 0.18 | 0 | 0.08 |
| 1 | 0.31 | 0.23 | 0.15 | 0.69 | 0.86 | 0.69 | 0.51 | 0.68 |
| 1 | 0.41 | 0.08 | 0.04 | 0.18 | 0.20 | 0.10 | 0 | 0.24 |
| 1 | 0.42 | 0.17 | 0.07 | 0.13 | 0.12 | 0.14 | 0 | 0.01 |
| 1 | 0.43 | 0.24 | 0.53 | 0.08 | 0.10 | 0.17 | 0 | 0.05 |
| 1 | 0.43 | 0.13 | 0.06 | 0.08 | 0.14 | 0.04 | 0 | 0.05 |
| 1 | 0.53 | 0.16 | 0.02 | 0.14 | 0.11 | 0.07 | 0 | 0.17 |
| 1 | 0.56 | 0.09 | 0.15 | 0.06 | 0.17 | 0.34 | 0.01 | 0.32 |
| 1 | 0.79 | 0.21 | 0.04 | 0.07 | 0.05 | 0.28 | 0 | 0.06 |
| 1 | 0.84 | 0.60 | 0.85 | 0.80 | 0.62 | 0.22 | 0.04 | 0.21 |
| 1 | 1.00 | 0.59 | 0.71 | 0.77 | 0.70 | 0.37 | 0.05 | 0.52 |
| 3 | 0 | 0 | 0 | 0 | 0 | 0.03 | 0 | 0 |
| 3 | 0 | 0.15 | 0 | 0.02 | 0 | 0.10 | 0 | 0.12 |
| 3 | 0 | 0.08 | 0 | 0 | 0 | 0.01 | 0 | 0.01 |
| 3 | 0.01 | 0.27 | 0 | 0.01 | 0 | 0.05 | 0 | 0 |
| 3 | 0.02 | 0.40 | 0 | 0 | 0 | 0.01 | 0 | 0 |
| 3 | 0.03 | 0.17 | 0.01 | 0.01 | 0 | 0.04 | 0 | 0.01 |
| 3 | 0.04 | 0.62 | 0.02 | 0.02 | 0.01 | 0.16 | 0 | 0.03 |
| 3 | 0.07 | 0.76 | 0.06 | 0.11 | 0.08 | 0.14 | 0 | 0.02 |
| 3 | 0.08 | 0.87 | 0.02 | 0.19 | 0.02 | 0.23 | 0 | 0.04 |
| 3 | 0.08 | 0.68 | 0.06 | 0.17 | 0.05 | 0.10 | 0.01 | 0.04 |
| 3 | 0.11 | 0.11 | 0.02 | 0.09 | 0.03 | 0.04 | 0 | 0.04 |
| 3 | 0.13 | 0.67 | 0.07 | 0.02 | 0.04 | 0.24 | 0.01 | 0.03 |
| 3 | 0.13 | 0.74 | 0.01 | 0.08 | 0 | 0.10 | 0 | 0.16 |
| 3 | 0.13 | 0.10 | 0.04 | 0.27 | 0.48 | 0.39 | 0 | 0.43 |
| 3 | 0.17 | 0.19 | 0 | 0.45 | 0.66 | 0.45 | 0.03 | 0.50 |
| 3 | 0.21 | 0.96 | 0.09 | 0.24 | 0.19 | 0.16 | 0 | 0.07 |
| 3 | 0.23 | 0.77 | 0.11 | 0.22 | 0.02 | 0.24 | 0 | 0.10 |
| 3 | 0.30 | 0.77 | 0.11 | 0.07 | 0.16 | 0.21 | 0 | 0.06 |
| 3 | 0.35 | 0.34 | 0.17 | 0.12 | 0.16 | 0.18 | 0 | 0.02 |
| 3 | 0.36 | 0.78 | 0.22 | 0.44 | 0.03 | 0.19 | 0 | 0.06 |
| 3 | 0.37 | 0.73 | 0.47 | 0.18 | 0.27 | 0.36 | 0 | 0.10 |
| 3 | 0.42 | 0.82 | 0.25 | 0.16 | 0.09 | 0.49 | 0.01 | 0.36 |
| 3 | 0.44 | 0.47 | 0.09 | 0.35 | 0.23 | 0.26 | 0.02 | 0.21 |
| 3 | 0.48 | 0.89 | 0.50 | 0.58 | 0.15 | 0.66 | 0 | 0.21 |

| S5 Table, continued. | | |  |  |  |  |  |  |
| --- | --- | --- | --- | --- | --- | --- | --- | --- |
| 3 | 0.53 | 0.38 | 0.15 | 0.29 | 0.10 | 0.29 | 0.02 | 0.21 |
| 3 | 0.55 | 0.93 | 0.25 | 0.49 | 0.33 | 0.27 | 0 | 0.06 |
| 3 | 0.58 | 0.76 | 0.59 | 0.59 | 0.62 | 0.66 | 0.10 | 0.27 |
| 3 | 0.65 | 0.68 | 0.06 | 0.19 | 0.18 | 0.34 | 0.01 | 0.18 |
| 3 | 0.68 | 0.68 | 0.26 | 0.24 | 0.37 | 0.26 | 0.07 | 0.15 |
| 3 | 0.91 | 0.52 | 0.23 | 0.67 | 0.19 | 0.18 | 0.01 | 0.07 |
| 4 | 0.07 | 0.10 | 0 | 0.06 | 0.23 | 0.38 | 0.18 | 0.08 |
| 4 | 0.09 | 0.10 | 0.04 | 0.03 | 0.16 | 0.09 | 0 | 0.01 |
| 4 | 0.12 | 0.32 | 0.03 | 0.01 | 0.03 | 0.03 | 0 | 0.06 |
| 4 | 0.12 | 0.05 | 0.08 | 0.03 | 0 | 0.02 | 0 | 0.01 |
| 4 | 0.13 | 0.13 | 0.13 | 0.30 | 0.01 | 0.04 | 0 | 0.03 |
| 4 | 0.21 | 0.13 | 0.10 | 0.03 | 0.01 | 0.07 | 0.02 | 0.05 |
| 4 | 0.29 | 0.21 | 0.32 | 0.17 | 0.07 | 0.09 | 0.03 | 0.19 |
| 4 | 0.29 | 0.10 | 0.36 | 0.51 | 0.31 | 0.13 | 0.01 | 0.05 |
| 4 | 0.30 | 0.22 | 0.11 | 0.64 | 0.07 | 0.57 | 0.13 | 0.23 |
| 4 | 0.30 | 0.40 | 0.71 | 0.56 | 0.12 | 0.20 | 0.02 | 0.06 |
| 4 | 0.31 | 0.33 | 0.46 | 0.21 | 0.16 | 0.25 | 0.02 | 0.14 |
| 4 | 0.39 | 0.36 | 0.85 | 0.76 | 0.47 | 0.33 | 0.03 | 0.09 |
| 4 | 0.40 | 0.18 | 0.28 | 0.26 | 0.13 | 0.16 | 0 | 0.05 |
| 4 | 0.41 | 0.45 | 0.72 | 0.64 | 0.32 | 0.05 | 0.02 | 0.10 |
| 4 | 0.42 | 0.76 | 0.99 | 0.57 | 0.37 | 0.53 | 0.02 | 0.18 |
| 4 | 0.51 | 0.23 | 0.35 | 0.36 | 0.05 | 0.26 | 0.03 | 0.38 |
| 4 | 0.53 | 0.56 | 0.23 | 0.53 | 0.07 | 0.12 | 0.03 | 0.10 |
| 4 | 0.55 | 0.08 | 0.46 | 0.52 | 0.41 | 0.50 | 0 | 0.55 |
| 4 | 0.58 | 0.31 | 0.60 | 0.22 | 0.29 | 0.61 | 0.03 | 0.14 |
| 4 | 0.66 | 0.84 | 0.93 | 0.77 | 0.65 | 0.36 | 0.01 | 0.13 |
| 4 | 0.70 | 0.23 | 0.31 | 0.41 | 0.45 | 0.22 | 0.03 | 0.16 |
| 4 | 0.76 | 0.18 | 0.05 | 0.50 | 0.17 | 0.13 | 0 | 0.10 |
| 4 | 0.90 | 0.81 | 0.97 | 0.46 | 0.56 | 0.43 | 0.37 | 0.39 |
| 6 | 0 | 0 | 0 | 0.01 | 0 | 0.22 | 0.01 | 0.01 |
| 6 | 0.05 | 0.01 | 0 | 0.24 | 0.05 | 0.48 | 0.32 | 0.13 |
| 6 | 0.06 | 0.05 | 0 | 0.36 | 0.32 | 0.32 | 0.42 | 0.29 |
| 6 | 0.06 | 0.08 | 0 | 0.08 | 0.36 | 0.32 | 0.08 | 0.12 |
| 6 | 0.09 | 0.05 | 0 | 0.34 | 0.21 | 0.35 | 0.38 | 0.21 |
| 6 | 0.13 | 0.06 | 0.01 | 0.34 | 0.12 | 0.13 | 0 | 0.18 |
| 6 | 0.14 | 0.20 | 0 | 0.11 | 0.62 | 0.65 | 0.16 | 0.12 |
| 6 | 0.17 | 0.11 | 0.11 | 0.36 | 0.12 | 0.26 | 0 | 0.06 |
| 6 | 0.19 | 0.04 | 0.02 | 0.26 | 0.03 | 0.07 | 0 | 0.09 |
| 6 | 0.21 | 0.15 | 0.73 | 0.01 | 0.03 | 0.10 | 0 | 0.05 |
| 6 | 0.23 | 0.26 | 0.17 | 0.41 | 0.18 | 0.40 | 0 | 0.21 |
| 6 | 0.24 | 0.01 | 0 | 0.05 | 0.03 | 0.09 | 0 | 0.06 |
| 6 | 0.27 | 0.30 | 0.29 | 0.45 | 0.12 | 0.19 | 0 | 0.07 |
| 6 | 0.34 | 0.22 | 0.14 | 0.51 | 0.72 | 0.57 | 0.02 | 0.42 |
| 6 | 0.35 | 0.09 | 0.01 | 0.60 | 0.03 | 0.13 | 0 | 0.15 |
| 6 | 0.45 | 0.17 | 0.05 | 0.44 | 0.22 | 0.11 | 0.01 | 0.22 |
| 6 | 0.57 | 0.20 | 0.19 | 0.66 | 0.26 | 0.30 | 0 | 0.42 |
| 6 | 0.60 | 0.07 | 0.02 | 0.77 | 0.03 | 0.29 | 0 | 0.07 |
| S5 Table, continued. | | |  |  |  |  |  |  |
| 6 | 0.73 | 0.16 | 0.22 | 0.52 | 0.05 | 0.38 | 0.08 | 0.58 |
| 6 | 0.77 | 0.83 | 0.80 | 0.32 | 0.30 | 0.14 | 0 | 0.06 |
| 7 | 0.01 | 0 | 0 | 0.04 | 0.02 | 0.28 | 0 | 0.21 |
| 7 | 0.02 | 0.06 | 0.01 | 0.14 | 0.79 | 0.38 | 0.02 | 0.09 |
| 7 | 0.02 | 0.04 | 0 | 0.39 | 0.62 | 0.32 | 0.01 | 0.05 |
| 7 | 0.04 | 0 | 0 | 0 | 0.01 | 0.02 | 0 | 0.01 |
| 7 | 0.04 | 0.14 | 0.01 | 0.38 | 0.83 | 0.30 | 0.01 | 0.05 |
| 7 | 0.04 | 0.03 | 0 | 0.07 | 0.05 | 0.45 | 0.30 | 0.06 |
| 7 | 0.05 | 0.02 | 0.01 | 0.03 | 0.07 | 0.11 | 0 | 0.01 |
| 7 | 0.06 | 0.20 | 0.03 | 0.44 | 0.11 | 0.80 | 0.26 | 0.06 |
| 7 | 0.07 | 0.23 | 0.04 | 0.07 | 0.50 | 0.34 | 0.02 | 0.05 |
| 7 | 0.08 | 0.05 | 0 | 0.05 | 0.57 | 0.26 | 0.01 | 0.17 |
| 7 | 0.08 | 0.21 | 0 | 0.33 | 0.66 | 0.46 | 0.01 | 0.26 |
| 7 | 0.09 | 0.18 | 0.02 | 0.33 | 0.75 | 0.29 | 0 | 0.08 |
| 7 | 0.09 | 0.07 | 0 | 0.23 | 0.14 | 0.14 | 0 | 0.08 |
| 7 | 0.09 | 0.07 | 0 | 0.13 | 0.27 | 0.25 | 0.32 | 0.22 |
| 7 | 0.09 | 0.29 | 0.02 | 0.29 | 0.55 | 0.51 | 0 | 0.27 |
| 7 | 0.10 | 0.04 | 0.01 | 0.02 | 0.06 | 0.10 | 0.06 | 0.10 |
| 7 | 0.10 | 0.05 | 0.01 | 0.05 | 0.36 | 0.09 | 0 | 0.05 |
| 7 | 0.12 | 0.11 | 0.01 | 0.02 | 0.22 | 0.02 | 0 | 0.02 |
| 7 | 0.12 | 0.05 | 0.01 | 0.47 | 0.49 | 0.27 | 0.01 | 0.17 |
| 7 | 0.12 | 0.01 | 0.06 | 0.31 | 0.25 | 0.29 | 0 | 0.08 |
| 7 | 0.12 | 0.16 | 0.05 | 0.36 | 0.40 | 0.72 | 0.15 | 0.31 |
| 7 | 0.15 | 0.16 | 0.10 | 0.70 | 0.60 | 0.20 | 0.01 | 0.21 |
| 7 | 0.17 | 0.11 | 0.07 | 0.07 | 0.75 | 0.49 | 0.83 | 0.52 |
| 7 | 0.17 | 0.01 | 0 | 0.05 | 0.02 | 0.15 | 0.17 | 0.19 |
| 7 | 0.19 | 0.09 | 0.01 | 0.12 | 0.35 | 0.13 | 0.29 | 0.23 |
| 7 | 0.19 | 0.04 | 0 | 0.18 | 0.05 | 0.23 | 0.03 | 0.36 |
| 7 | 0.20 | 0.04 | 0 | 0.43 | 0.32 | 0.33 | 0.51 | 0.18 |
| 7 | 0.21 | 0.19 | 0.02 | 0.22 | 0.37 | 0.18 | 0 | 0.08 |
| 7 | 0.21 | 0.21 | 0.05 | 0.11 | 0.34 | 0.22 | 0.03 | 0.08 |
| 7 | 0.38 | 0.05 | 0.04 | 0.43 | 0.31 | 0.18 | 0.02 | 0.14 |
| 7 | 0.39 | 0.16 | 0.06 | 0.14 | 0.95 | 0.43 | 0.08 | 0.22 |
| 7 | 0.40 | 0.49 | 0.14 | 0.81 | 0.67 | 0.89 | 0.30 | 0.46 |
| 7 | 0.55 | 0.19 | 0.02 | 0.45 | 0.56 | 0.26 | 0.33 | 0.29 |
| 7 | 0.65 | 0.19 | 0.17 | 0.25 | 0.70 | 0.29 | 0.01 | 0.36 |
| 7 | 0.67 | 0.27 | 0.06 | 0.29 | 0.63 | 0.42 | 0.03 | 0.12 |
| 7 | 0.68 | 0.07 | 0.06 | 0.12 | 0.19 | 0.03 | 0.01 | 0.29 |
| 7 | 0.69 | 0.39 | 0.60 | 0.51 | 0.74 | 0.44 | 0.01 | 0.57 |
| 8 | 0 | 0 | 0 | 0 | 0 | 0.01 | 0 | 0 |
| 8 | 0 | 0 | 0 | 0 | 0 | 0 | 0 | 0 |
| 8 | 0.01 | 0.51 | 0.14 | 0.06 | 0.40 | 0.23 | 0.12 | 0.13 |
| 8 | 0.02 | 0 | 0 | 0.03 | 0.01 | 0.11 | 0 | 0.09 |
| 8 | 0.03 | 0.07 | 0.01 | 0.45 | 0.23 | 0.73 | 0.06 | 0.24 |
| 8 | 0.04 | 0.09 | 0 | 0.06 | 0.03 | 0.44 | 0.02 | 0.31 |
| 8 | 0.04 | 0.14 | 0.02 | 0.66 | 0.26 | 0.54 | 0.03 | 0.20 |

| S5 Table, continued. | | |  |  |  |  |  |  |
| --- | --- | --- | --- | --- | --- | --- | --- | --- |
| 8 | 0.07 | 0.10 | 0 | 0.07 | 0.27 | 0.34 | 0.02 | 0.25 |
| 8 | 0.08 | 0.50 | 0 | 0.02 | 0.27 | 0.21 | 0.01 | 0.04 |
| 8 | 0.09 | 0.05 | 0.01 | 0.21 | 0.47 | 0.93 | 0.88 | 0.33 |
| 8 | 0.12 | 0.01 | 0 | 0.07 | 0.06 | 0.48 | 0 | 0.08 |
| 8 | 0.12 | 0.09 | 0.03 | 0.53 | 0.90 | 0.95 | 0.08 | 0.28 |
| 8 | 0.14 | 0.01 | 0 | 0.08 | 0.09 | 0.71 | 0.02 | 0.10 |
| 8 | 0.14 | 0.07 | 0.09 | 0.82 | 0.25 | 0.87 | 0.61 | 0.45 |
| 8 | 0.14 | 0.21 | 0.10 | 0.73 | 0.24 | 0.86 | 0.01 | 0.27 |
| 8 | 0.14 | 0.10 | 0.01 | 0.06 | 0.04 | 0.52 | 0 | 0.03 |
| 8 | 0.15 | 0.10 | 0.01 | 0.31 | 0.15 | 0.64 | 0.62 | 0.16 |
| 8 | 0.18 | 0.31 | 0.02 | 0.08 | 0.11 | 0.82 | 0.03 | 0.08 |
| 8 | 0.21 | 0.10 | 0.03 | 0.45 | 0.52 | 0.41 | 0.90 | 0.56 |
| 8 | 0.21 | 0.10 | 0.02 | 0.66 | 0.01 | 0.24 | 0.01 | 0.06 |
| 8 | 0.22 | 0.04 | 0 | 0.45 | 0.13 | 0.26 | 0.01 | 0.22 |
| 8 | 0.32 | 0.01 | 0 | 0.19 | 0.06 | 0.13 | 0 | 0.11 |
| 8 | 0.39 | 0.55 | 0.06 | 0.23 | 0.29 | 0.12 | 0.01 | 0.06 |
| 8 | 0.87 | 0.76 | 0.09 | 0.94 | 0.98 | 0.79 | 0.90 | 0.89 |
| 9 | 0.01 | 0.01 | 0 | 0.02 | 0.07 | 0.43 | 0.37 | 0.04 |
| 9 | 0.01 | 0.01 | 0 | 0 | 0.11 | 0.04 | 0.04 | 0.03 |
| 9 | 0.02 | 0.01 | 0 | 0.02 | 0.03 | 0.34 | 0.27 | 0.21 |
| 9 | 0.03 | 0.01 | 0 | 0.16 | 0.21 | 0.50 | 0.88 | 0.41 |
| 9 | 0.03 | 0 | 0 | 0.14 | 0.32 | 0.42 | 0.86 | 0.19 |
| 9 | 0.03 | 0.03 | 0 | 0.03 | 0.20 | 0.12 | 0.21 | 0.33 |
| 9 | 0.03 | 0.01 | 0 | 0.12 | 0.13 | 0.22 | 0.20 | 0.12 |
| 9 | 0.03 | 0.01 | 0 | 0.07 | 0.11 | 0.17 | 0.25 | 0.06 |
| 9 | 0.04 | 0.01 | 0.01 | 0.03 | 0.12 | 0.10 | 0.13 | 0.06 |
| 9 | 0.05 | 0.01 | 0 | 0.20 | 0.65 | 0.37 | 0.44 | 0.20 |
| 9 | 0.06 | 0.06 | 0.04 | 0.54 | 0.95 | 0.87 | 0.91 | 0.32 |
| 9 | 0.06 | 0.01 | 0 | 0.20 | 0.18 | 0.22 | 0.52 | 0.34 |
| 9 | 0.07 | 0.08 | 0 | 0.89 | 0.40 | 0.45 | 0.18 | 0.39 |
| 9 | 0.08 | 0.02 | 0.01 | 0.09 | 0.03 | 0.17 | 0.04 | 0.43 |
| 9 | 0.08 | 0.11 | 0 | 0.08 | 0.16 | 0.40 | 0.18 | 0.07 |
| 9 | 0.08 | 0.06 | 0 | 0.17 | 0.38 | 0.32 | 0.19 | 0.12 |
| 9 | 0.09 | 0.05 | 0 | 0.18 | 0.06 | 0.25 | 0.18 | 0.12 |
| 9 | 0.09 | 0.04 | 0.01 | 0.06 | 0.22 | 0.48 | 0.16 | 0.36 |
| 9 | 0.09 | 0.45 | 0.09 | 0.25 | 0.66 | 0.71 | 0.82 | 0.27 |
| 9 | 0.10 | 0.04 | 0 | 0.02 | 0.49 | 0.29 | 0.49 | 0.13 |
| 9 | 0.12 | 0.09 | 0.02 | 0.42 | 0.25 | 0.62 | 0.89 | 0.41 |
| 9 | 0.12 | 0.01 | 0 | 0.07 | 0.32 | 0.65 | 0.92 | 0.70 |
| 9 | 0.14 | 0.01 | 0 | 0.25 | 0.13 | 0.30 | 0.19 | 0.58 |
| 9 | 0.14 | 0.01 | 0 | 0.25 | 0.58 | 0.69 | 0.53 | 0.62 |
| 9 | 0.14 | 0.01 | 0.01 | 0.15 | 0.12 | 0.28 | 0.48 | 0.27 |
| 9 | 0.16 | 0.08 | 0 | 0.23 | 0.21 | 0.37 | 0.26 | 0.18 |
| 9 | 0.17 | 0.11 | 0.01 | 0.67 | 0.47 | 0.70 | 0.68 | 0.20 |
| 9 | 0.17 | 0.18 | 0.03 | 0.06 | 0.04 | 0.17 | 0 | 0.12 |
| 9 | 0.18 | 0.25 | 0.02 | 0.24 | 0.37 | 0.56 | 0.23 | 0.16 |

| S5 Table, continued. | | |  |  |  |  |  |  |
| --- | --- | --- | --- | --- | --- | --- | --- | --- |
| 9 | 0.20 | 0.01 | 0 | 0.08 | 0.29 | 0.55 | 0.42 | 0.06 |
| 9 | 0.21 | 0.02 | 0.02 | 0.27 | 0.17 | 0.46 | 0.50 | 0.21 |
| 9 | 0.23 | 0.06 | 0 | 0.20 | 0.23 | 0.19 | 0.38 | 0.19 |
| 9 | 0.25 | 0.16 | 0.01 | 0.69 | 0.31 | 0.84 | 0.74 | 0.31 |
| 9 | 0.26 | 0.06 | 0.01 | 0.60 | 0.26 | 0.27 | 0.12 | 0.56 |
| 9 | 0.27 | 0.09 | 0.03 | 0.07 | 0.29 | 0.27 | 0.39 | 0.12 |
| 9 | 0.37 | 0.15 | 0.05 | 0.27 | 0.69 | 0.46 | 0.36 | 0.26 |
| 9 | 0.45 | 0.18 | 0 | 0.49 | 0.32 | 0.81 | 0.83 | 0.29 |
| 9 | 0.71 | 0.18 | 0.01 | 0.53 | 0.54 | 0.55 | 0.43 | 0.34 |
| 9 | 0.84 | 0.57 | 0.29 | 0.98 | 0.99 | 0.96 | 0.97 | 0.91 |
| 9 | 0.85 | 0.35 | 0.29 | 0.55 | 0.36 | 0.80 | 0.29 | 0.17 |
| 10 | 0 | 0 | 0 | 0 | 0 | 0 | 0 | 0.07 |
| 10 | 0 | 0 | 0 | 0 | 0 | 0 | 0 | 0.05 |
| 10 | 0 | 0.01 | 0 | 0.02 | 0.06 | 0.26 | 0.47 | 0 |
| 10 | 0 | 0 | 0 | 0 | 0 | 0.01 | 0 | 0.44 |
| 10 | 0.01 | 0.04 | 0 | 0.02 | 0 | 0.03 | 0 | 0.02 |
| 10 | 0.01 | 0 | 0 | 0 | 0 | 0.03 | 0 | 0.78 |
| 10 | 0.01 | 0.02 | 0 | 0.06 | 0.14 | 0.55 | 0.04 | 0.40 |
| 10 | 0.02 | 0 | 0 | 0 | 0 | 0 | 0 | 0.33 |
| 10 | 0.02 | 0 | 0 | 0 | 0 | 0.01 | 0 | 0.51 |
| 10 | 0.04 | 0 | 0 | 0.02 | 0 | 0.05 | 0 | 0.93 |
| 10 | 0.06 | 0.01 | 0 | 0.36 | 0.54 | 0.32 | 0.79 | 0.07 |
| 10 | 0.07 | 0 | 0 | 0.08 | 0.03 | 0.22 | 0.01 | 0.25 |
| 10 | 0.07 | 0 | 0 | 0 | 0 | 0.02 | 0 | 0.68 |
| 10 | 0.07 | 0.01 | 0 | 0.12 | 0.04 | 0.12 | 0 | 0.86 |
| 10 | 0.08 | 0 | 0 | 0.04 | 0.06 | 0.13 | 0 | 0.74 |
| 10 | 0.12 | 0.01 | 0 | 0.14 | 0.13 | 0.44 | 0.10 | 0.93 |
| 10 | 0.18 | 0.05 | 0 | 0.07 | 0.10 | 0.04 | 0 | 0.45 |
| 10 | 0.31 | 0.01 | 0 | 0.04 | 0.01 | 0.15 | 0 | 0.01 |
| 10 | 0.32 | 0.07 | 0.01 | 0.71 | 0.68 | 0.90 | 0.99 | 0.62 |
| 10 | 0.42 | 0.04 | 0.05 | 0.29 | 0.47 | 0.48 | 0.01 | 0.67 |
| 10 | 0.75 | 0.24 | 0.18 | 0.67 | 0.21 | 0.38 | 0.03 | 0.22 |
| 10 | 0.89 | 0.15 | 0.58 | 0.36 | 0.13 | 0.13 | 0.03 | 0.04 |
