# Supplementary material for: Genetic Differentiation, Isolation-by-Distance, and Metapopulation Dynamics of the Arizona Treefrog (Hyla wrightorum) in an Isolated Portion of Its Range
Source: PLoS One. 2016 Aug 9;11(8):e0160655. doi: 10.1371/journal.pone.0160655 (PMC4978385; doi:10.1371/journal.pone.0160655)
Supplement: S6 Table — (DOCX) [file pone.0160655.s007.docx]

| S6 Table. Delta K calculations and log likelihoods for Structure output with LOCPRIOR. | | | | | | |
| --- | --- | --- | --- | --- | --- | --- |
| HYWR: all individuals: | | | | | | |
| # K | Reps | Mean LnP(K) | Stdev LnP(K) | Ln'(K) | \|Ln''(K)\| | Delta K |
| 1 | 10 | -11959.15 | 0.40 | NA | NA | NA |
| **2** | **10** | **-11576.89** | **9.01** | **382.26** | **180.20** | **20.01** |
| **3** | **10** | **-11374.83** | **5.21** | **202.06** | **99.93** | **19.19** |
| 4 | 10 | -11272.70 | 24.36 | 102.13 | 57.39 | 2.36 |
| 5 | 10 | -11227.96 | 13.40 | 44.74 | 17.58 | 1.31 |
| 6 | 10 | -11200.80 | 15.73 | 27.16 | 55.88 | 3.55 |
| 7 | 10 | -11229.52 | 51.43 | -28.72 | 5.40 | 0.11 |
| 8 | 10 | -11263.64 | 60.67 | -34.12 | NA | NA |
|  |  |  |  |  |  |  |
| Following K = 3 for all individuals (results visualized in Figure 3) | | | | | | |
| HYWR 1: Populations 1, 2, 3, and 4: | | | | | | |
| # K | Reps | Mean LnP(K) | Stdev LnP(K) | Ln'(K) | \|Ln''(K)\| | Delta K |
| 1 | 10 | -3973.01 | 0.64 | NA | NA | NA |
| **2** | **10** | **-3928.04** | **5.29** | **44.97** | **61.43** | **11.60** |
| 3 | 10 | -3944.50 | 28.02 | -16.46 | 1.22 | 0.04 |
| 4 | 10 | -3959.74 | 71.62 | -15.24 | 47.82 | 0.67 |
| 5 | 10 | -4022.80 | 50.85 | -63.06 | NA | NA |
|  |  |  |  |  |  |  |
| HYWR 1.1: Populations 1, 2, and 4: | | | | | | |
| # K | Reps | Mean LnP(K) | Stdev LnP(K) | Ln'(K) | \|Ln''(K)\| | Delta K |
| 1 | 10 | -2413.62 | 0.68 | NA | NA | NA |
| 2 | 10 | -2398.15 | 16.78 | 15.47 | 6.33 | 0.38 |
| **3** | **10** | **-2376.35** | **20.11** | **21.80** | **118.76** | **5.90** |
| 4 | 10 | -2473.31 | 74.17 | -96.96 | NA | NA |
|  |  |  |  |  |  |  |
| HYWR 2: Populations 6, 7, 8, and 9: | | | | | | |
| # K | Reps | Mean LnP(K) | Stdev LnP(K) | Ln'(K) | \|Ln''(K)\| | Delta K |
| 1 | 10 | -6377.88 | 0.41 | NA | NA | NA |
| **2** | **10** | **-6251.28** | **2.36** | **126.60** | **128.97** | **54.59** |
| 3 | 10 | -6253.65 | 8.42 | -2.37 | 31.64 | 3.76 |
| 4 | 10 | -6287.66 | 28.90 | -34.01 | 155.91 | 5.39 |
| 5 | 10 | -6477.58 | 231.96 | -189.92 | NA | NA |
|  |  |  |  |  |  |  |
| HYWR 2.1: Populations 6, 7, and 8: | | | | | | |
| # K | Reps | Mean LnP(K) | Stdev LnP(K) | Ln'(K) | \|Ln''(K)\| | Delta K |
| **1** | **10** | **-4315.63** | **0.54** | **NA** | **NA** | **NA** |
| 2 | 10 | -4348.21 | 40.25 | -32.58 | 5.43 | 0.13 |
| 3 | 10 | -4375.36 | 51.64 | -27.15 | 86.83 | 1.68 |
| 4 | 10 | -4489.34 | 160.65 | -113.98 | NA | NA |

| S6 Table, continued. | | | | | | |
| --- | --- | --- | --- | --- | --- | --- |
| Following K = 2 for all individuals | | | | | | |
| HYWR (B) 1: Populations 1, 2, 3, and 4: | | | | | | |
| # K | Reps | Mean LnP(K) | Stdev LnP(K) | Ln'(K) | \|Ln''(K)\| | Delta K |
| 1 | 10 | -3973.01 | 0.64 | NA | NA | NA |
| **2** | **10** | **-3928.04** | **5.29** | **44.97** | **61.43** | **11.60** |
| 3 | 10 | -3944.50 | 28.02 | -16.46 | 1.22 | 0.04 |
| 4 | 10 | -3959.74 | 71.62 | -15.24 | 47.82 | 0.67 |
| 5 | 10 | -4022.80 | 50.85 | -63.06 | NA | NA |
|  |  |  |  |  |  |  |
| HYWR (B) 1.1: Populations 1, 2, and 4: | | | |  |  |  |
| # K | Reps | Mean LnP(K) | Stdev LnP(K) | Ln'(K) | \|Ln''(K)\| | Delta K |
| 1 | 10 | -2413.62 | 0.68 | NA | NA | NA |
| 2 | 10 | -2398.15 | 16.78 | 15.47 | 6.33 | 0.38 |
| **3** | **10** | **-2376.35** | **20.11** | **21.80** | **118.76** | **5.90** |
| 4 | 10 | -2473.31 | 74.17 | -96.96 | NA | NA |
|  |  |  |  |  |  |  |
| HYWR (B) 2: Populations 6, 7, 8, 9, and 10: | | | |  |  |  |
| # K | Reps | Mean LnP(K) | Stdev LnP(K) | Ln'(K) | \|Ln''(K)\| | Delta K |
| 1 | 10 | -7685.34 | 0.37 | NA | NA | NA |
| 2 | 10 | -7507.39 | 0.93 | 177.95 | 59.71 | 64.43 |
| **3** | **10** | **-7389.15** | **2.57** | **118.24** | **189.06** | **73.64** |
| 4 | 10 | -7459.97 | 33.94 | -70.82 | 105.75 | 3.12 |
| 5 | 10 | -7425.04 | 20.34 | 34.93 | 163.07 | 8.02 |
| 6 | 10 | -7553.18 | 35.66 | -128.14 | NA | NA |
|  |  |  |  |  |  |  |
| HYWR (B) 2.1: Populations 6, 7, and 8: | | | |  |  |  |
| # K | Reps | Mean LnP(K) | Stdev LnP(K) | Ln'(K) | \|Ln''(K)\| | Delta K |
| 1 | 10 | **-4315.80** | **0.47** | **NA** | **NA** | **NA** |
| 2 | 10 | -4327.26 | 6.63 | -11.46 | 71.02 | 10.72 |
| 3 | 10 | -4409.74 | 21.38 | -82.48 | 9.62 | 0.45 |
| 4 | 10 | -4501.84 | 31.78 | -92.10 | NA | NA |
