# Supplementary material for: Genetic Differentiation, Isolation-by-Distance, and Metapopulation Dynamics of the Arizona Treefrog (Hyla wrightorum) in an Isolated Portion of Its Range
Source: PLoS One. 2016 Aug 9;11(8):e0160655. doi: 10.1371/journal.pone.0160655 (PMC4978385; doi:10.1371/journal.pone.0160655)
Supplement: S7 Table — (DOCX) [file pone.0160655.s008.docx]

| S7 Table. Delta K calculations and log liklihoods for Structure output without LOCPRIOR. | | | | | | |
| --- | --- | --- | --- | --- | --- | --- |
| HYWR: all individuals: | | | | | | |
| # K | Reps | Mean LnP(K) | Stdev LnP(K) | Ln'(K) | \|Ln''(K)\| | Delta K |
| 1 | 10 | -11959.23 | 0.15 | NA | NA | NA |
| **2** | **10** | **-11565.05** | **2.37** | **394.18** | **325.66** | **137.31** |
| 3 | 10 | -11496.53 | 16.35 | 68.52 | 87.84 | 5.37 |
| 4 | 10 | -11340.17 | 5.25 | 156.36 | 43.07 | 8.20 |
| 5 | 10 | -11226.88 | 3.28 | 113.29 | 49.13 | 14.99 |
| 6 | 10 | -11162.72 | 4.67 | 64.16 | 205.61 | 44.00 |
| 7 | 10 | -11304.17 | 83.22 | -141.45 | 147.37 | 1.77 |
| 8 | 10 | -11298.25 | 56.41 | 5.92 | 18.56 | 0.33 |
| 9 | 10 | -11273.77 | 34.52 | 24.48 | NA | NA |
|  |  |  |  |  |  |  |
| HYWR 1: Populations 1, 2, 3, and 4: | | | | | | |
| # K | Reps | Mean LnP(K) | Stdev LnP(K) | Ln'(K) | \|Ln''(K)\| | Delta K |
| **1** | **10** | **-3972.75** | **0.46** | **NA** | **NA** | **NA** |
| 2 | 10 | -4020.65 | 14.89 | -47.90 | 9.62 | 0.65 |
| 3 | 10 | -4078.17 | 94.81 | -57.52 | 134.54 | 1.42 |
| 4 | 10 | -4001.15 | 90.14 | 77.02 | 157.39 | 1.75 |
| 5 | 10 | -4081.52 | 15.19 | -80.37 | NA | NA |
|  |  |  |  |  |  |  |
| HYWR 2: Populations 6, 7, 8, 9, and 10: | | | |  |  |  |
| # K | Reps | Mean LnP(K) | Stdev LnP(K) | Ln'(K) | \|Ln''(K)\| | Delta K |
| 1 | 10 | -7737.21 | 0.28 | NA | NA | NA |
| 2 | 10 | -7561.19 | 1.16 | 176.02 | 57.15 | 49.15 |
| **3** | **10** | **-7442.32** | **2.00** | **118.87** | **180.61** | **90.11** |
| 4 | 10 | -7504.06 | 18.37 | -61.74 | 98.83 | 5.38 |
| 5 | 10 | -7466.97 | 27.54 | 37.09 | 164.73 | 5.98 |
| 6 | 10 | -7594.61 | 77.24 | -127.64 | NA | NA |
|  |  |  |  |  |  |  |
| HYWR 2.1: Populations 6, 7, and 8: | | | |  |  |  |
| # K | Reps | Mean LnP(K) | Stdev LnP(K) | Ln'(K) | \|Ln''(K)\| | Delta K |
| 1 | 10 | **-4368.13** | **0.58** | **NA** | **NA** | **NA** |
| 2 | 10 | -4384.11 | 17.90 | -15.98 | 113.23 | 6.33 |
| 3 | 10 | -4513.32 | 89.95 | -129.21 | 53.12 | 0.59 |
| 4 | 10 | -4589.41 | 169.40 | -76.09 | NA | NA |
