# Supplementary material for: Genetic Differentiation, Isolation-by-Distance, and Metapopulation Dynamics of the Arizona Treefrog (Hyla wrightorum) in an Isolated Portion of Its Range
Source: PLoS One. 2016 Aug 9;11(8):e0160655. doi: 10.1371/journal.pone.0160655 (PMC4978385; doi:10.1371/journal.pone.0160655)
Supplement: S8 Table — (DOCX) [file pone.0160655.s009.docx]

| S8 Table. Landscape data - sources, description, and details. | | | | |
| --- | --- | --- | --- | --- |
|  | Canopy | Streams | Slope | Distance |
| Details | 2001 NLCD canopy density dataset. | NHDPlus Version 2, downloaded from National Map Viewer (USGS). | Calculated with 9-m Digital Elevation Model from National Elevation Dataset. | Uniform resistance layer. |
| Resolution | 30 m | 100 m shapefile buffer around streams, converted to raster with resolution of 30 m | 9 m, resampled to 30 m | 30 m |
| Resistance scale | 1 - 100: inversely scaled to % canopy cover (0-100%) | 1 or 100: binary - 1 (low resistance) for riparian buffer area, 100 for non-riparian area | 1 - 100: inversely related to % slope | 1: uniform throughout |
| Source | <http://www.mrlc.gov/nlcd01_data.php> | <http://viewer.nationalmap.gov/viewer/> | <http://ned.usgs.gov/downloads.asp> |  |
