# Supplementary material for: Genetic Differentiation, Isolation-by-Distance, and Metapopulation Dynamics of the Arizona Treefrog (Hyla wrightorum) in an Isolated Portion of Its Range
Source: PLoS One. 2016 Aug 9;11(8):e0160655. doi: 10.1371/journal.pone.0160655 (PMC4978385; doi:10.1371/journal.pone.0160655)
Supplement: S9 Table — (DOCX) [file pone.0160655.s010.docx]

| S9 Table. Pearson correlation coefficients between all distance matrices (genetic and spatial). | | | | | |
| --- | --- | --- | --- | --- | --- |
|  | *F_ST_*/(1-_FST_) | *D_ps_* | Canopy | Stream | Slope |
| *D_ps_* | 0.964 |  |  |  |  |
| Canopy | 0.083 | 0.212 |  |  |  |
| Stream | 0.093 | 0.171 | 0.005 |  |  |
| Slope | 0.385 | 0.340 | -0.660 | 0.050 |  |
| Null | 0.357 | 0.502 | 0.756 | 0.075 | -0.045 |
