# Supplementary material for: Genetic Differentiation, Isolation-by-Distance, and Metapopulation Dynamics of the Arizona Treefrog (Hyla wrightorum) in an Isolated Portion of Its Range
Source: PLoS One. 2016 Aug 9;11(8):e0160655. doi: 10.1371/journal.pone.0160655 (PMC4978385; doi:10.1371/journal.pone.0160655)
Supplement: S10 Table — (DOCX) [file pone.0160655.s011.docx]

| S10 Table. Resistances between populations for landscape and spatial variables. | | | | | | | |
| --- | --- | --- | --- | --- | --- | --- | --- |
| Distance - uniform resistance | | | | | | | |
| Pops | 1 | 3 | 4 | 6 | 7 | 8 | 9 |
| 3 | 1.082 |  |  |  |  |  |  |
| 4 | 1.020 | 1.016 |  |  |  |  |  |
| 6 | 1.086 | 0.981 | 0.916 |  |  |  |  |
| 7 | 1.155 | 0.970 | 1.047 | 0.968 |  |  |  |
| 8 | 1.174 | 0.988 | 1.072 | 1.001 | 0.775 |  |  |
| 9 | 1.165 | 1.044 | 1.028 | 0.939 | 0.946 | 0.973 |  |
| 10 | 1.278 | 1.156 | 1.153 | 1.094 | 1.058 | 1.058 | 1.002 |
| Slope | | | | | | | |
| Pops | 1 | 3 | 4 | 6 | 7 | 8 | 9 |
| 3 | 15.15 |  |  |  |  |  |  |
| 4 | 8.85 | 16.63 |  |  |  |  |  |
| 6 | 9.25 | 15.77 | 9.41 |  |  |  |  |
| 7 | 12.48 | 18.10 | 13.47 | 11.68 |  |  |  |
| 8 | 14.60 | 20.21 | 15.73 | 14.14 | 11.74 |  |  |
| 9 | 10.66 | 17.29 | 11.35 | 9.38 | 11.67 | 14.09 |  |
| 10 | 9.82 | 16.65 | 10.67 | 9.26 | 11.22 | 13.34 | 8.20 |
| Canopy | | | | | | | |
| Pops | 1 | 3 | 4 | 6 | 7 | 8 | 9 |
| 3 | 63.72 |  |  |  |  |  |  |
| 4 | 78.36 | 51.46 |  |  |  |  |  |
| 6 | 78.21 | 44.98 | 60.49 |  |  |  |  |
| 7 | 72.79 | 34.08 | 58.91 | 50.92 |  |  |  |
| 8 | 67.55 | 28.86 | 53.76 | 45.96 | 28.37 |  |  |
| 9 | 83.34 | 47.92 | 67.89 | 59.23 | 50.58 | 45.21 |  |
| 10 | 101.00 | 64.72 | 86.40 | 79.05 | 66.85 | 60.35 | 75.47 |
| Stream | | | | | | | |
| Pops | 1 | 3 | 4 | 6 | 7 | 8 | 9 |
| 3 | 14.73 |  |  |  |  |  |  |
| 4 | 40.81 | 44.71 |  |  |  |  |  |
| 6 | 12.42 | 15.21 | 39.05 |  |  |  |  |
| 7 | 36.34 | 35.49 | 65.35 | 34.86 |  |  |  |
| 8 | 46.43 | 44.28 | 75.64 | 45.34 | 57.59 |  |  |
| 9 | 14.88 | 15.14 | 43.82 | 13.19 | 28.38 | 39.93 |  |
| 10 | 44.97 | 44.41 | 74.08 | 43.64 | 57.96 | 67.41 | 36.66 |
